# Benchmarking Hybrid CNN‐Transformer Versus Pure Transformer Architectures for Accelerated Hyperpolarized ^129^Xe MRI Reconstruction

**DOI:** 10.1002/jmri.70314

**Published:** 2026-03-27

**Authors:** Ramtin Babaeipour, Matthew S. Fox, Grace Parraga, Alexei Ouriadov

**Affiliations:** ^1^ School of Biomedical Engineering, Faculty of Engineering The University of Western Ontario London Ontario Canada; ^2^ Department of Physics and Astronomy The University of Western Ontario London Ontario Canada; ^3^ Lawson Research Institute London Ontario Canada; ^4^ Department of Medical Biophysics The University of Western Ontario London Ontario Canada; ^5^ Robarts Research Institute The University of Western Ontario London Ontario Canada

**Keywords:** asthma, COPD, deep learning, hyperpolarized ^129^Xe MRI, long‐COVID, lung imaging, medical imaging, MRI reconstruction, pulmonary imaging, Vision Transformers

## Abstract

**Background:**

Hyperpolarized ^129^Xe MRI faces technical challenges including low signal‐to‐noise ratio and breath‐hold constraints. Current literature focuses on proprietary deep learning methods or image‐domain enhancements.

**Purpose:**

To present a comprehensive evaluation of transformer and hybrid CNN‐transformer architectures integrating dual‐domain (k‐space and image) processing for HP ^129^Xe MRI reconstruction.

**Study Type:**

Retrospective.

**Population:**

Two hundred five participants (22 healthy [male and female, 18–85 years], 26 COPD [male and female, 50–85 years], 90 asthma [male and female, 18–70 years], 67 long‐COVID [male and female, 18–70 years]) yielding 1640 2D slices. Dataset split: 80% training (1312 slices), 10% validation (164 slices), 10% test (164 slices).

**Field Strength/Sequence:**

3 T; 3D fast gradient‐recalled echo.

**Assessment:**

Five architectures were compared: KTMR (hybrid transformer‐CNN), KIKI‐net (pure CNN), ReconFormer, SwinMR, and MR‐IPT (pure transformer) at acceleration factors of 3, 7, and 10. Performance was assessed using peak signal‐to‐noise ratio (PSNR), structural similarity index measure (SSIM), and normalized mean squared error (NMSE). Ventilation defect percentage (VDP) agreement with semi‐automated analysis was evaluated.

**Statistical Tests:**

Friedman test with post hoc Dunn's test and Benjamini‐Hochberg correction for multiple comparisons. Significance level: *p* < 0.05.

**Results:**

At 10‐fold acceleration, KTMR produced PSNR of 36.4 ± 2.8 dB and SSIM of 0.88 ± 0.12, significantly outperforming KIKI‐net (32.5 ± 3.4 dB, 0.81 ± 0.12), ReconFormer (29.7 ± 2.6 dB, 0.76 ± 0.12), SwinMR (30.5 ± 2.8 dB, 0.76 ± 0.09), and MR‐IPT (28.8 ± 2.4 dB, 0.74 ± 0.11). VDP measurements showed mean bias of 1.94% at 3‐fold, 2.12% at 7‐fold, and 2.69% at 10‐fold acceleration.

**Data Conclusion:**

KTMR demonstrated superior performance for HP ^129^Xe MRI reconstruction at high acceleration factors.

**Evidence Level:**

3.

**Technical Efficacy:**

Stage 1.

## Plain Language Summary

Hyperpolarized ^129^Xe MRI is a specialized lung imaging technique that allows doctors to visualize how air moves through the lungs, helping diagnose conditions like asthma, COPD, and long‐COVID. However, the scan requires breath‐holding and uses expensive gas. This study tested five artificial intelligence methods to reconstruct high‐quality lung images from faster, reduced data acquisitions. Using data from 205 patients, it was found that a hybrid approach combining convolutional neural networks and transformers produced the best image quality, even at very high acceleration. This could reduce breath‐hold times from 10 to 2–3 s, making the scan more accessible for vulnerable patients.

## 1 Introduction

Hyperpolarized ^129^Xe magnetic resonance imaging (HP ^129^Xe MRI) has emerged as a powerful noninvasive technique for assessing pulmonary microstructure and gas exchange function, offering insights into lung pathophysiology across various respiratory diseases including chronic obstructive pulmonary disease (COPD), asthma, and post‐COVID‐19 pulmonary complications [1]. Unlike conventional proton MRI, HP ^129^Xe MRI directly visualizes the inhaled gas distribution within the airspaces, providing functional ventilation information that complements structural imaging modalities [2].

Despite its clinical potential, widespread adoption of HP ^129^Xe MRI faces several technical challenges. The high cost of isotopically enriched ^129^Xe and the finite, rapidly decaying polarization impose constraints on acquisition efficiency, making it critical to maximize the signal‐to‐noise ratio (SNR) obtained per unit of administered gas. While current consortium‐level protocols achieve whole‐lung coverage within approximately 10 s, further improvements in SNR efficiency through advanced reconstruction could enable the use of lower xenon doses or natural abundance xenon, potentially reducing per‐scan isotope costs by approximately 10‐fold [3]. Additionally, reduced acquisition requirements could shorten breath‐holds for pediatric patients and those with severe respiratory compromise who cannot tolerate standard protocols [4]. In HP ^129^Xe MRI, the SNR is directly proportional to the degree of polarization of the gas and, if sufficiently high, can be exchanged for shorter acquisition times (and shorter breath‐holds) while holding image quality constant. Furthermore, the finite and rapidly decaying polarization of ^129^Xe imposes strict temporal constraints on data acquisition protocols [5].

Traditional acceleration techniques for MRI such as parallel imaging [6] and compressed sensing have shown promise for HP ^129^Xe imaging but often suffer from residual artifacts and noise amplification that can compromise diagnostic quality [7, 8, 9, 10, 11]. The emergence of deep learning‐based reconstruction methods has affected medical imaging, offering superior artifact suppression and noise reduction when compared to conventional reconstruction approaches [12]. These methods have demonstrated success in accelerating various MRI applications, from cardiac imaging [13, 14] to neuroimaging [15], by learning complex mappings between undersampled and fully sampled data.

Deep learning methods developed for HP gas MRI have shown strong performance in segmentation and analysis tasks [16, 17, 18, 19, 20, 21, 22]. In the area of reconstruction, deep learning reconstruction approaches have exclusively relied on convolutional neural network (CNN) architectures. In 2019, Duan et al. [23] implemented the deep learning framework for HP ^129^Xe MRI reconstruction, introducing a cascaded CNN approach (CasNet) comprised of coarse and fine networks (C‐net and F‐net) that incorporated anatomical prior knowledge from proton MRI to achieve 4‐fold acceleration, outperforming compressed sensing methods while preserving ventilation defect percentage accuracy. Building upon this foundation, subsequent work developed deep cascaded residual dense networks (DC‐RDN) [24] specifically for accelerating multiple *b*‐value HP gas diffusion MRI, demonstrating that CNN architectures could maintain accurate lung morphometry measurements while reducing breath‐hold times at four‐fold acceleration, highlighting the potential for CNN‐based approaches in specialized HP gas applications beyond simple ventilation imaging. More recently, Stewart et al. [25] explored the application of commercial deep learning reconstruction pipelines (AIR Recon DL) originally developed for conventional MRI to HP ^129^Xe imaging, showing that even nonspecialized CNN approaches could significantly improve SNR by more than three‐fold and enable imaging with natural‐abundance xenon, though with notable biases in quantitative ventilation metrics due to edge sharpening effects associated with the super‐resolution training objective. While these CNN‐based methods have demonstrated substantial improvements in reconstruction speed and image quality, their local receptive fields (typically 3 × 3 or 5 × 5 pixels per layer) may limit their ability to capture spatially distributed ventilation patterns spanning the entire lung field, such as gravitational ventilation gradients, lobar‐level defect distributions, or correlations between anatomically distant regions of airway obstruction. This could result in reconstructions that accurately recover local texture but fail to maintain physiologically consistent global ventilation patterns. Complementary approaches that capture long‐range spatial dependencies and global ventilation patterns could help address these limitations, particularly in distributed lung pathophysiology. Previous research has established a minimum clinically important difference (MCID) of 2% for ^129^Xe VDP in asthma patients [26], providing a critical benchmark for evaluating clinical acceptability of accelerated reconstructions.

Recent advances in deep learning architectures have opened new possibilities for MRI reconstruction, particularly in the development of Vision Transformers (ViTs) and their adaptations for medical imaging [27, 28, 29, 30, 31, 32, 33, 34, 35, 36, 37]. Transformer architectures excel at capturing long‐range dependencies and global context, which may be particularly advantageous for HP ^129^Xe MRI, spatial correlations across the lung parenchyma including gravitational ventilation gradients, lobar ventilation heterogeneity, the spatial extent and boundaries of ventilation defects reflecting regional airway obstruction, and anterior–posterior signal variations influenced by body position contain diagnostically important information that benefits from reconstruction methods capable of modeling these long‐range dependencies. Moreover, the integration of k‐space data consistency constraints within deep learning frameworks has shown superior performance compared to image‐domain‐only approaches, as it preserves the fundamental physics of MRI acquisition [38].

However, despite the success of transformer architectures in conventional MRI reconstruction and other medical imaging applications, their potential for HP ^129^Xe MRI reconstruction remains unexplored.

The aim of this proof‐of‐concept study is to systematically evaluate and benchmark hybrid CNN–transformer, pure CNN, and pure transformer architectures for accelerated HP ^129^Xe static ventilation MRI reconstruction across a range of clinically relevant acceleration factors (AFs). Specifically, the study examines whether integrating dual‐domain processing with explicit k‐space data consistency constraints improves reconstruction quality compared with image‐domain‐only approaches. In addition, the clinical utility of accelerated reconstructions is assessed by quantifying agreement in ventilation defect percentage measurements relative to fully sampled reference images.

## 2 Materials and Methods

### 2.1 Study Population and Data Acquisition

All participants provided written informed consent, and the study protocols were approved by a local institutional review board and Health Canada (ex‐smokers with and without COPD [NCT02279329, male and female, aged 50–85 years of age with a clinical diagnosis of COPD or Bronchiectais or > 10 pack/year smoking history]), asthma (NCT04651777 [male and female aged 18–70 years of age with a clinical diagnosis of eosinophilic asthma], NCT02263794 [male and female aged 18–60 years of age with a clinical diagnosis of asthma], NCT02351141 [male and female aged 18–60 with a clinical diagnosis of asthma] and NCT03733535 [male and female aged 18–70 with a clinical diagnosis of asthma]), long‐COVID‐19 (NCT04584671 [male and female aged 18–70 with documented by positive COVID‐19 test and/or clinical history of mild or severe COVID‐19 infection]), and healthy volunteers (NCT02484885 [male and female aged 18–85 with stable health on the basis of medical history]). A total of 205 participants were retrospectively evaluated from a single institution, comprising 22 healthy individuals, 26 patients with COPD, 90 patients with asthma, and 67 patients with long‐COVID‐19 syndrome.

### 2.2 MRI Acquisition Protocol

All imaging was performed on a 3 T Discovery MR750 scanner (GE Healthcare, WI, USA) equipped with specialized hardware for hyperpolarized gas imaging. The system utilized a flexible vest quadrature coil (Clinical MR Solutions, Milwaukee, WI) and custom‐built rigid asymmetric quadrature birdcage coil optimized for ^129^Xe detection. Hyperpolarized ^129^Xe gas was produced using a model 9820 polarizer (Polarean, Durham, NC, USA) achieving polarization levels of 10%–40%.

Hyperpolarized ^129^Xe ventilation imaging was performed using a three‐dimensional Fast Gradient‐Recalled Echo (FGRE) sequence with the following parameters: field of view (FOV) = 400 × 400 × 240 mm^3^; matrix = 80 × 128 × 16 (x‐direction partial echo acquisition); bandwidth = 32 kHz; repetition time/echo time (TR/TE) = 6.7/1.5 ms; and a variable flip‐angle RF excitation scheme.

The gas administration protocol was standardized across participants. For ^1^H imaging, participants inhaled 1.0 L of N_2_ at functional residual capacity (FRC) to provide a state of lung inflation (volume) comparable to that of the hyperpolarized gas acquisition. For ^129^Xe imaging, participants inhaled a gas mixture containing 400 mL hyperpolarized ^129^Xe and 600 mL ^4^He (total volume 1.0 L) at FRC, followed by a 15‐s breath‐hold during image acquisition to minimize motion artifacts and ensure complete data collection within the temporal constraints imposed by T_1_ relaxation of the ^129^Xe gas.

### 2.3 Data Preprocessing and Undersampling Strategy

**FIGURE 1:**
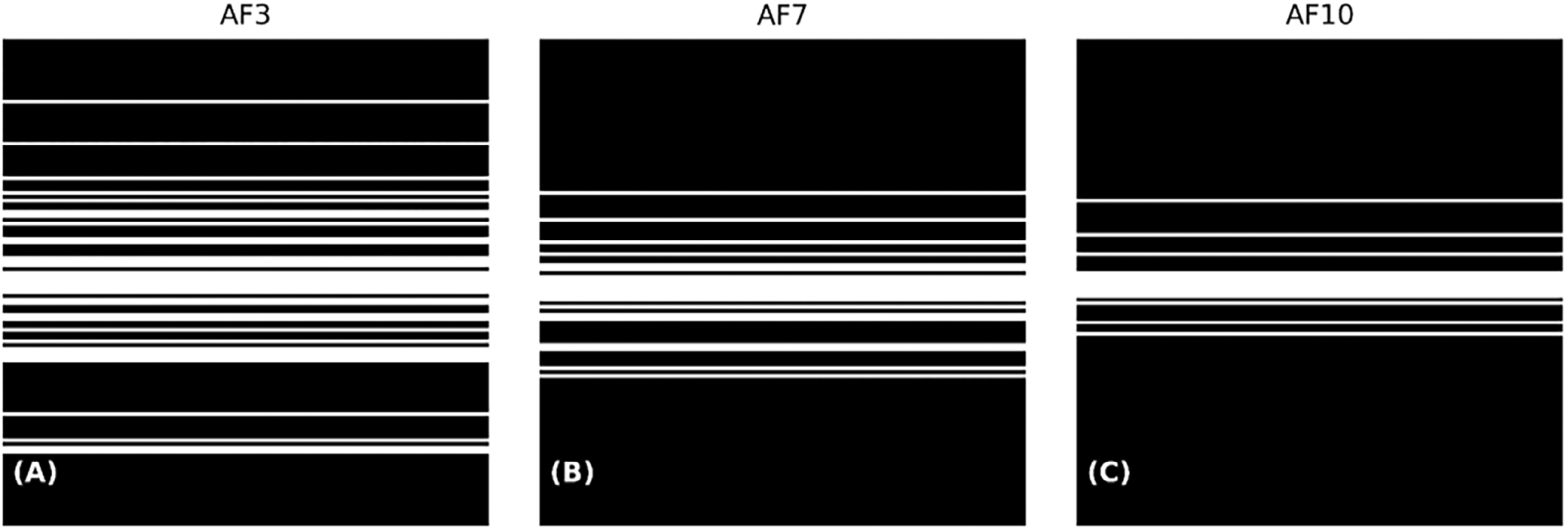
Retrospective k‐space undersampling masks used for acceleration factor simulation. Representative undersampling patterns applied in the phase‐encoding direction for (A) AF = 3, (B) AF = 7, and (C) AF = 10 applied in the phase‐encoding direction. White lines indicate sampled k‐space locations, whereas black regions represent unsampled locations. The masks employ variable‐density Cartesian undersampling, with denser sampling in the central k‐space region (low spatial frequencies) and sparser sampling in the periphery (high spatial frequencies) to optimize reconstruction quality.

From the acquired dataset, 1640 2D slices were reconstructed and resampled to a standardized matrix size of 128 × 128 voxels. To simulate accelerated acquisition scenarios, three compressed sensing undersampling masks employing variable density Cartesian sampling [38] patterns were generated, corresponding to AFs of 3, 7, and 10 (Figure 1).

It is important to note that the original k‐space data had already been accelerated with an AF of 1.6 due to the partial echo acquisition factor of 62.5% in the readout direction. The AFs of 3, 7, and 10 reported throughout this study refer specifically to additional retrospective undersampling applied in the phase‐encoding (Y) direction only. When combined with the existing 1.6‐fold acceleration in the readout direction, the actual total AFs relative to fully sampled k‐space are approximately 4.8, 11.2, and 16 for the reported AF 3, 7, and 10 conditions, respectively. This combined acceleration approach reflects realistic clinical acquisition scenarios where partial echo techniques are commonly employed to reduce acquisition times in HP ^129^Xe MRI [39].

For model training and evaluation, the dataset was stratified based on slice position and participant demographics to ensure representative distributions across the training (80%), validation (10%), and test (10%) sets. Care was taken to prevent data leakage by keeping all slices from the same participant within the same training, validation, or test dataset.

### 2.4 Deep Learning Architectures

Five distinct deep learning architectures were implemented and systematically evaluated for HP ^129^Xe MRI reconstruction. These included both state‐of‐the‐art transformer‐based approaches and established CNN methodologies. Each architecture was carefully adapted for the unique characteristics of hyperpolarized gas imaging.

Accurate reconstruction of HP ^129^Xe ventilation MRI must preserve several critical imaging features: (1) sharp boundaries of ventilation defects, which define the spatial extent of airway obstruction and are essential for VDP quantification; (2) signal homogeneity within ventilated lung regions, where subtle intensity variations may indicate partial obstruction or gravitational gradients; (3) low‐frequency ventilation patterns spanning the entire lung field, such as anterior–posterior or superior–inferior gradients that reflect gravitational and structural influences on gas distribution; and (4) fine‐scale texture within ventilated regions that may carry information about regional gas distribution heterogeneity. These requirements guided the selection of five architectures spanning three paradigms: KIKI‐net (pure CNN) was selected for its dual‐domain k‐space and image processing with explicit data consistency; KTMR (hybrid CNN‐transformer) for combining local CNN feature extraction with global transformer attention and k‐space constraints; and SwinMR, ReconFormer, and MR‐IPT (pure transformers) for their respective approaches to capturing long‐range spatial dependencies, hierarchical windowed attention, recurrent multi‐scale processing, and prompt‐guided multi‐task reconstruction.

### 2.5 KIKI‐Net (K‐Space Image Iterative Network)

KIKI‐net [40] implements a cross‐domain CNN architecture that iteratively alternates between k‐space and image domain processing. The architecture addresses the ill‐posed inverse problem of MRI reconstruction by formulating separate optimization objectives for k‐space completion and image restoration, connected through interleaved data consistency (IDC) operations that enforce physics‐based constraints by replacing network‐predicted k‐space values at sampled locations with original acquired measurements.

The k‐space CNN (KCNN) processes complex‐valued k‐space data through a feature extraction network, a 25‐layer inference network for k‐space completion, and a reconstruction network. The image CNN (ICNN) mirrors this structure but operates on complex image data with residual skip connections. The complete architecture alternates between K‐net (KCNN with inverse Fourier transform) and I‐net (ICNN with data consistency) operations, enabling progressive refinement across domains. Training is performed incrementally, with each 25‐layer sub‐network trained independently using domain‐specific loss functions due to the computational complexity of the combined 100‐layer architecture.

### 2.6 SwinMR (Swin Transformer for MRI Reconstruction)

SwinMR [41] leverages the Swin Transformer [28] architecture with hierarchical feature learning through shifted windowing mechanisms, specifically adapted for medical image reconstruction. The architecture represents a significant advancement in applying ViTs to MRI reconstruction by addressing the computational limitations of traditional multi‐head self‐attention while maintaining global context modeling capabilities. Unlike conventional CNNs, which are constrained by local receptive fields, or standard transformers, which require prohibitive computational costs for full global attention, SwinMR employs shifted window‐based multi‐head self‐attention. This mechanism processes images in non‐overlapping local windows while enabling cross‐window communication through alternating shifted windowing patterns. This approach reduces computational complexity from quadratic to linear scaling while preserving the ability to capture long‐range spatial dependencies crucial for MRI reconstruction.

The SwinMR framework consists of three primary modules: an input module (IM), a feature extraction module (FEM), and an output module (OM). The IM and OM are implemented as 2D convolutional layers for dimensional mapping, while the FEM comprises cascaded Residual Swin Transformer Blocks (RSTBs) that contain multiple Swin Transformer Layers (STLs) with alternating windowed and shifted‐windowed multi‐head self‐attention mechanisms. Each RSTB incorporates patch embedding and unembedding operations with residual connections to facilitate stable training and faster convergence. SwinMR employs a sophisticated multi‐domain loss function that operates across spatial, frequency, and perceptual domains, combining pixel‐wise Charbonnier loss for robustness, frequency‐domain loss to ensure k‐space consistency, which is essential for MRI physics, and perceptual VGG loss to enhance visual quality while preserving reconstruction fidelity.

SwinMR's parallel imaging capability, incorporating multi‐coil sensitivity maps into data processing and loss computation, enables more accurate texture and detail preservation compared to single‐channel approaches. The robustness validation across different masks and noise conditions, along with the downstream brain tumor segmentation experiment on the BraTS17 dataset, confirms that SwinMR reconstructions maintain diagnostic quality suitable for clinical applications. Segmentation performance is nearly equivalent to that of ground truth images [41].

### 2.7 KTMR (K‐Space Transformer MRI Reconstruction)

KTMR [42] combines the representational power of Swin Transformers with explicit k‐space data consistency constraints, creating a hybrid architecture that leverages both attention mechanisms and physics‐based constraints for superior reconstruction quality. The architecture maintains a three‐module structure consisting of a convolution‐transformer feature extraction module, a reconstruction module, and a data consistency layer. Unlike conventional CNN‐based approaches that are limited by local receptive fields, KTMR employs Swin Transformer blocks to capture long‐range dependencies and global context which are essential for MRI reconstruction. The framework is trained as a single unified network that takes undersampled images as input and directly produces reconstructed images as output without requiring separate training stages, while incorporating k‐space data consistency by replacing predicted k‐space values at sampled locations with original measurements.

The feature extraction module begins with a convolutional layer for early visual processing and low‐level feature extraction, followed by six RSTBs, each containing six STLs and ending with a final convolutional layer. The Swin Transformer components utilize shifted‐window‐based multi‐head self‐attention with a window size of 8 × 8, 180 embedding dimensions, and 6 attention heads, enabling efficient computation while maintaining global context modeling capabilities. The reconstruction module employs two convolutional layers to restore spatial details and generate the final reconstructed image. The critical innovation lies in the integration of a k‐space data consistency layer that enforces physics‐informed constraints by replacing predicted k‐space values with original measurements at sampled locations, ensuring reconstruction fidelity and preserving learned features from unsampled regions.

Comprehensive experimental validation across multiple anatomies (brain, heart, knee) and different MRI contrasts (T1‐weighted, Simultaneous Non‐contrast Angiography [SNAP] [43]) demonstrates KTMR's superior performance over traditional compressed sensing methods, dictionary learning approaches, and CNN‐based alternatives [42]. The architecture shows particular strength at high AFs, achieving optimal peak signal‐to‐noise ratio (PSNR) and structural similarity index measure (SSIM) values with 10% sampling ratios where traditional methods struggle significantly. The k‐space data consistency layer proves essential for maintaining structural integrity and improving reconstruction quality, with ablation studies confirming its positive impact across different imaging scenarios. KTMR's effectiveness stems from the synergy between transformer‐based global feature learning and physics‐based k‐space constraints, enabling high‐quality reconstruction while achieving approximately 10‐fold acceleration in acquisition speed compared to fully sampled protocols.

### 2.8 MR‐IPT (Multi‐Prompt Image Processing Transformer)

MR‐IPT [44] implements a sophisticated prompt‐guided transformer architecture that unifies reconstruction across multiple AFs through learned prompt embeddings, marking a paradigm shift toward generalized MRI reconstruction frameworks. The architecture extends the Image Processing Transformer (IPT) paradigm by treating different undersampling reconstruction setups as distinct tasks, enabling a single unified model to handle multiple undersampling scenarios. MR‐IPT consists of five core components: heads for feature extraction from undersampled images, tails for image reconstruction, a prompt encoder that generates task‐specific embeddings based on acceleration labels, a shared encoder utilizing shifted‐window multi‐head self‐attention to capture global context, and a lightweight decoder incorporating prompt self‐attention and two‐way cross‐attention mechanisms. This design enables the core ViT backbone to focus on learning robust universal feature representations while the prompt system provides task‐specific conditioning for different reconstruction scenarios.

The framework implements three distinct variants to explore different aggregation strategies for handling diverse undersampling patterns. MR‐IPT‐type groups heads and tails based on acceleration ratios, with each head‐tail pair specializing in reconstructing images from different sampling masks within the same AF. MR‐IPT‐level aggregates heads and tails based on sampling masks, allowing each pair to generalize across different acceleration ratios for the same mask type. MR‐IPT‐split assigns a dedicated head‐tail pair to each unique combination of sampling mask and acceleration ratio, providing maximum task‐specific optimization. The shared encoder‐decoder architecture processes image patch tokens alongside prompt tokens generated by the prompt encoder, enabling effective feature refinement and reconstruction by integrating both visual and task‐specific information. The lightweight decoder design allows for a deeper encoder architecture without significantly increasing computational costs, thereby enhancing the model's representational capacity.

MR‐IPT employs large‐scale pre‐training on RadImageNet with diverse acceleration ratios and sampling patterns, including both 1D and 2D undersampling masks, to maximize generalization capabilities. This pre‐training strategy enables robust zero‐shot generalization to unseen sampling configurations during inference, even with limited downstream fine‐tuning data.

### 2.9 ReconFormer (Recurrent Transformer for MRI Reconstruction)

ReconFormer [45] represents a novel, recurrent transformer‐based architecture specifically designed for MRI reconstruction tasks. The method incorporates a locally pyramidal yet globally columnar structure that enables multi‐scale representation modeling at each processing stage while maintaining high‐resolution image details with exceptional accuracy.

The architecture consists of three recurrent units and a Refine Module (RM), employing a globally columnar structure to maintain high‐resolution information. Each recurrent unit contains an encoder, a ReconFormer block, and a decoder, with ReconFormer blocks operating on features at different scales (×0.5, ×1.0, and ×1.5) of the original image using adjustable‐stride convolution layers.

The core innovation is the Recurrent Pyramid Transformer Layer (RPTL) featuring Recurrent Scale‐wise Attention (RSA). Unlike standard multi‐head self‐attention, RSA consists of several attention scale heads (×1, ×3, and ×5) operating on multi‐scale patches in parallel. This enables efficient in‐place scale modeling and forms a feature pyramid by directly projecting features at various scales into multiple attention heads.

### 2.10 Training Configuration and Optimization

All models were implemented in PyTorch and trained on NVIDIA GPUs with CUDA acceleration to leverage parallel processing capabilities. Training hyperparameters were systematically optimized across different architectures to ensure a fair comparison and optimal performance for each model type. The optimization strategy employed the AdamW optimizer with a weight decay of 0.0001 to prevent overfitting while maintaining training stability [46]. Learning rates were carefully tuned for each architecture, ranging from 0.0001 to 0.0002 depending on the specific model requirements and complexity. Batch sizes were adjusted between 2 and 8 samples to accommodate varying model computational demands and memory constraints, with larger models using smaller batch sizes to fit within GPU memory constraints. Batch sizes were set to 2 for KTMR and SwinMR, 4 for ReconFormer and MR‐IPT, and 8 for KIKI‐net, adjusted to accommodate each model's memory requirements. While batch size differences can influence optimization dynamics and convergence behavior, all models were trained to convergence with early stopping based on validation performance, mitigating the potential impact of batch size variation on final reconstruction quality.

Training was conducted for up to 300 epochs with early stopping mechanisms implemented to prevent overfitting and reduce unnecessary computational overhead. Learning rate scheduling followed a ReduceLROnPlateau strategy with a patience of 10 epochs. The learning rate was reduced by a factor of 0.5 when validation performance plateaued, with a minimum threshold maintained to ensure continued training progression. Gradient clipping was applied with a maximum norm of 1.0 to stabilize training dynamics and to prevent exploding gradients, which is particularly important for transformer‐based architectures. Data augmentation strategies included horizontal flipping with a 20% probability and vertical flipping with a 10% probability to increase data diversity, along with elastic deformations and standardized resizing to 128 × 128 pixels to ensure consistent input dimensions across architectures.

Training stability was ensured through several measures, including NaN detection and handling during loss computation to prevent crashes, input validation to maintain data integrity, and mixed‐precision training where applicable to optimize memory usage and speed. These comprehensive training protocols ensured robust and reproducible model development across all evaluated architectures.

The experimental framework was implemented using PyTorch version 1.12 with CUDA 11.6 support, running on NVIDIA RTX 3090 graphics cards equipped with 24GB of VRAM to handle the computational demands of transformer‐based architectures. Training duration varied from 50 to 300 epochs depending on model convergence characteristics, with transformer‐based methods typically requiring longer training due to their higher parameter complexity. The validation strategy employed stratified cross‐validation to ensure representative data distribution across training and testing phases, which helps prevent potential bias from imbalanced dataset groups (i.e., training, validation, test).

Reproducibility was ensured through multiple measures, including fixed random seeds (set to 42) for all data splitting operations, which enabled consistent experimental results across multiple runs. Deterministic algorithms were employed wherever computationally feasible to minimize variability in training outcomes, though some operations necessarily remained non‐deterministic due to GPU optimization requirements. Comprehensive hyperparameter logging and model checkpointing were implemented in all experiments to enable result reproduction and facilitate detailed analysis of training dynamics. Standardized evaluation protocols were maintained across all architectures to ensure a fair comparison, with identical data preprocessing, evaluation metrics, and statistical analysis procedures consistently applied to all models.

### 2.11 Statistical Analysis

Model performance was quantitatively assessed using three well‐established metrics that provide complementary perspectives on reconstruction quality. PSNR measures the ratio between the maximum possible power of a signal and the power of corrupting noise, providing a measured, objective assessment of reconstruction fidelity in decibels. SSIM evaluates the perceived quality by comparing luminance, contrast, and structural information between reconstructed and reference images, providing a metric that is more aligned with human visual perception than simple pixel‐wise comparisons. Normalized mean squared error (NMSE) quantifies the relative reconstruction error by dividing the mean squared error by the energy of the ground truth image, providing a scale‐independent measure of reconstruction accuracy.

Statistical significance across different models and AFs was assessed using the non‐parametric Friedman test, chosen for its robustness to non‐normal distributions commonly encountered in medical imaging data. This approach enabled reliable comparison of multiple models across different experimental conditions without requiring assumptions about data distribution normality. Post hoc pairwise comparisons were performed using Dunn's test with Benjamini‐Hochberg correction for multiple comparisons to control the false discovery rate. This approach identifies specific significant differences between model pairs while maintaining statistical rigor across the comprehensive evaluation framework. Statistical significance was set at *p* < 0.05 for all analyses.

## 3 Results

Comprehensive evaluation of five deep learning architectures across three acceleration factors (AF = 3, 7, 10) revealed significant differences in reconstruction performance compared to the zero‐filled reconstruction baseline. Table 1 presents the quantitative results for all model‐AF combinations.

**TABLE 1:** Quantitative performance metrics across all models and acceleration factors.

Model	AF	PSNR (dB)	SSIM	NMSE
KTMR	3	38.11 ± 2.18	0.93 ± 0.05	0.01 ± 0.01
KTMR	7	37.11 ± 2.58	0.90 ± 0.09	0.01 ± 0.01
KTMR	10	36.41 ± 2.82	0.87 ± 0.11	0.02 ± 0.02
KIKI‐net	3	37.27 ± 4.23	0.90 ± 0.06	0.01 ± 0.01
KIKI‐net	7	33.98 ± 3.56	0.85 ± 0.10	0.02 ± 0.02
KIKI‐net	10	32.50 ± 3.39	0.81 ± 0.12	0.02 ± 0.02
ReconFormer	3	34.36 ± 3.52	0.85 ± 0.09	0.01 ± 0.01
ReconFormer	7	31.93 ± 3.03	0.81 ± 0.11	0.03 ± 0.02
ReconFormer	10	29.71 ± 2.56	0.76 ± 0.12	0.04 ± 0.03
SwinMR	3	30.62 ± 2.09	0.79 ± 0.04	0.03 ± 0.02
SwinMR	7	30.57 ± 2.33	0.76 ± 0.07	0.03 ± 0.02
SwinMR	10	30.53 ± 2.76	0.76 ± 0.09	0.03 ± 0.02
MR‐IPT	3	31.97 ± 2.60	0.83 ± 0.07	0.02 ± 0.01
MR‐IPT	7	30.77 ± 2.67	0.79 ± 0.11	0.03 ± 0.02
MR‐IPT	10	28.84 ± 2.39	0.74 ± 0.11	0.05 ± 0.02
Zero Filling	3	28.65 ± 1.92	0.76 ± 0.06	0.05 ± 0.02
Zero Filling	7	27.84 ± 1.96	0.70 ± 0.08	0.06 ± 0.02
Zero Filling	10	26.01 ± 1.82	0.64 ± 0.09	0.10 ± 0.03

The Friedman test revealed statistically significant differences among all reconstruction methods at each AF. Table 1 presents the complete quantitative results for all model–AF combinations.

At AF = 3, KTMR achieved the highest PSNR (38.11 ± 2.18 dB) and SSIM (0.93 ± 0.05), significantly outperforming KIKI‐net (37.27 ± 4.23 dB, 0.90 ± 0.06), ReconFormer (34.36 ± 3.52 dB, 0.85 ± 0.09), SwinMR (30.62 ± 2.09 dB, 0.79 ± 0.04), MR‐IPT (31.97 ± 2.60 dB, 0.83 ± 0.07), and zero‐filled reconstruction (28.65 ± 1.92 dB, 0.76 ± 0.06) on post hoc testing. The KTMR–KIKI‐net comparison for NMSE was not significant (*p* = 0.536). KIKI‐net significantly outperformed all remaining methods. All deep learning methods significantly outperformed zero‐filled reconstruction.

At AF = 7, KTMR maintained superior performance (PSNR: 37.11 ± 2.58 dB, SSIM: 0.90 ± 0.09) compared to KIKI‐net (33.98 ± 3.56 dB, 0.85 ± 0.10), ReconFormer (31.93 ± 3.03 dB, 0.81 ± 0.11), SwinMR (30.57 ± 2.33 dB, 0.76 ± 0.07), MR‐IPT (30.77 ± 2.67 dB, 0.79 ± 0.11), and zero‐filled reconstruction (27.84 ± 1.96 dB, 0.70 ± 0.08). The KTMR–KIKI‐net NMSE comparison approached but did not reach significance (*p* = 0.030). Among pure transformer methods, MR‐IPT and SwinMR did not differ significantly for PSNR (*p* = 0.288) or NMSE (*p* = 0.836), and MR‐IPT and ReconFormer did not differ for SSIM (*p* = 0.007). All deep learning methods significantly outperformed zero‐filled reconstruction.

**FIGURE 2:**
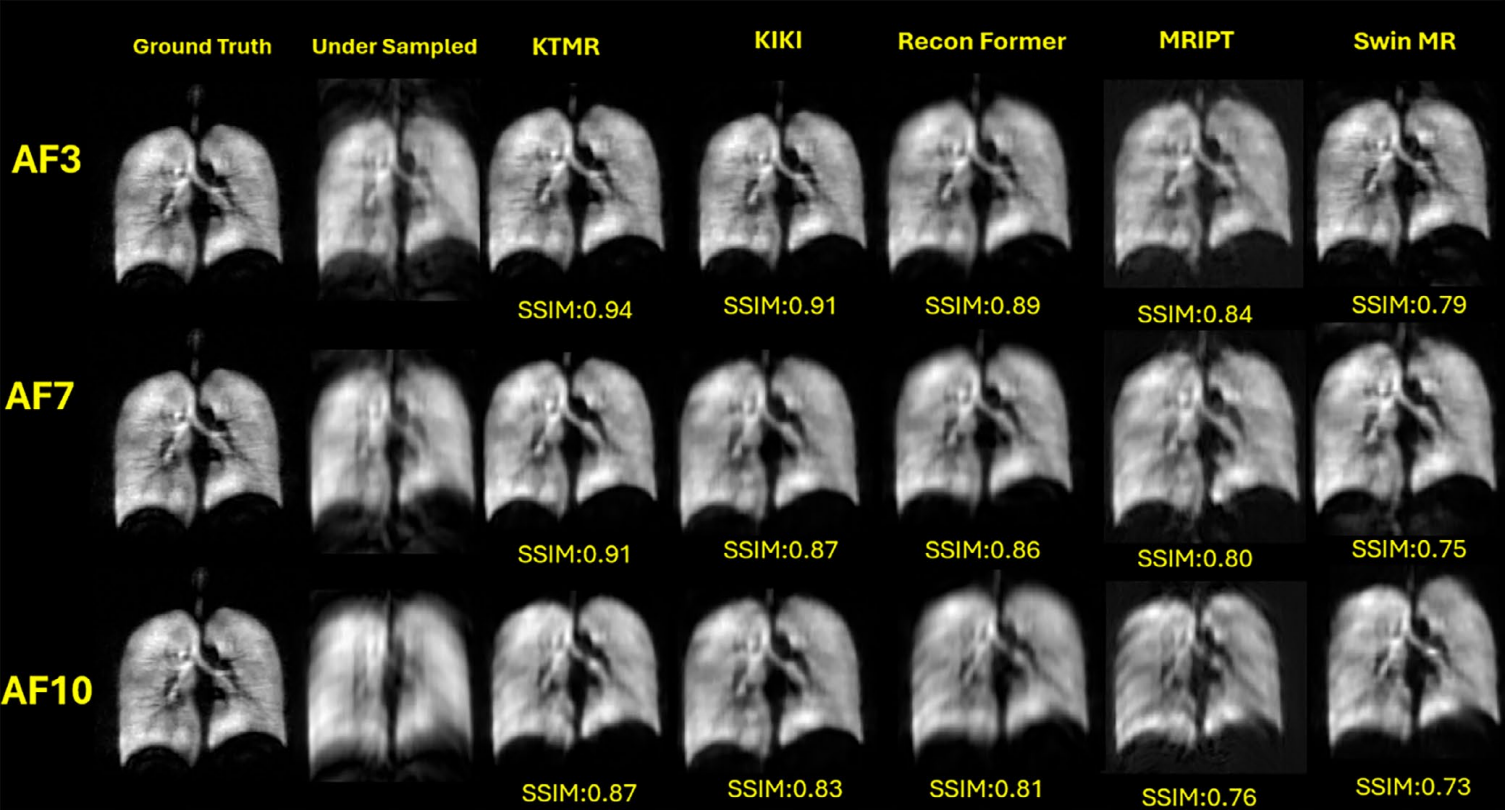
Qualitative comparison of HP ^**129**^Xe MRI reconstruction quality across five deep learning architectures for a representative patient with relatively uniform ventilation distribution. Reconstructions are shown for acceleration factors 3, 7, and 10 (AF3, AF7, AF10), compared with the ground truth and undersampled images. KTMR in most cases achieves the highest SSIM values (0.94, 0.91, 0.87) across acceleration factors, followed by KIKI‐net (0.91, 0.87, 0.83). Pure transformer methods show progressively lower performance. Observe the superior preservation of lung parenchyma detail and edge definition in KTMR reconstructions.

**FIGURE 3:**
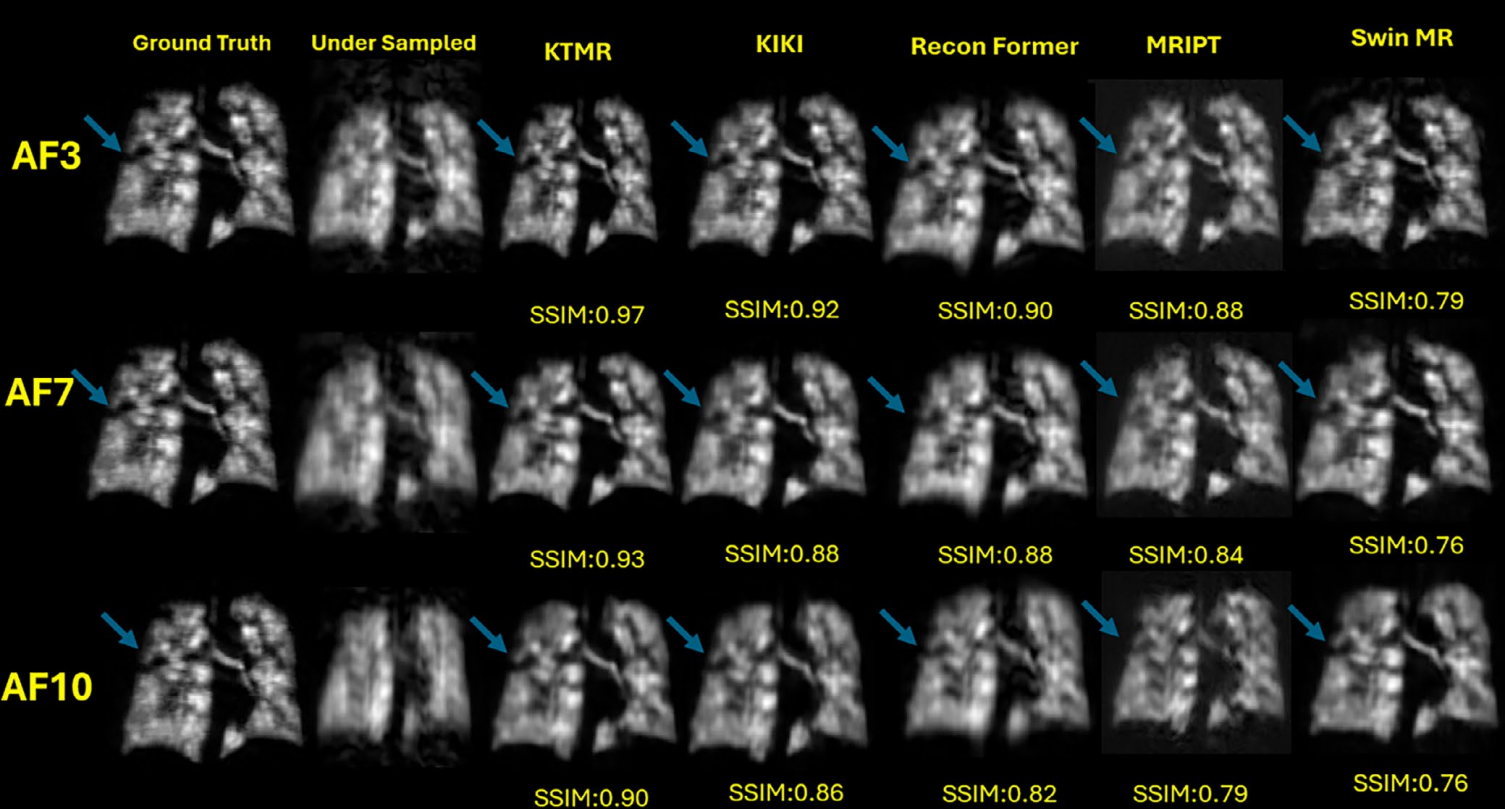
Qualitative comparison of HP ^**129**^Xe MRI reconstruction quality across five deep learning architectures for a representative patient with heterogeneous ventilation defects. Reconstructions are shown for acceleration factors 3, 7, and 10 (AF3, AF7, AF10), compared to ground truth and undersampled images. SSIM values are displayed for each reconstruction. KTMR demonstrates exceptional reconstruction fidelity (SSIM: 0.97, 0.93, 0.90) across all acceleration factors, with KIKI‐net showing competitive performance (0.92, 0.88, 0.86). Pure transformer approaches exhibit noticeable quality degradation, particularly at higher acceleration factors. The complex ventilation patterns and defect boundaries are best preserved by the hybrid CNN‐transformer architecture (KTMR). Blue arrows highlight small ventilation defects, illustrating how these critical features are preserved or degraded across reconstruction methods and acceleration factors.

At AF = 10, KTMR (PSNR: 36.41 ± 2.82 dB, SSIM: 0.87 ± 0.11) significantly outperformed all other methods, including KIKI‐net (32.50 ± 3.39 dB, 0.81 ± 0.12), ReconFormer (29.71 ± 2.56 dB, 0.76 ± 0.12), SwinMR (30.53 ± 2.76 dB, 0.76 ± 0.09), MR‐IPT (28.84 ± 2.39 dB, 0.74 ± 0.11), and zero‐filled reconstruction (26.01 ± 1.82 dB, 0.64 ± 0.09). KIKI‐net significantly outperformed all remaining methods. Among pure transformer methods, MR‐IPT and ReconFormer did not differ for NMSE (*p* = 0.008), MR‐IPT and SwinMR did not differ for SSIM (*p* = 0.137), and ReconFormer and SwinMR did not differ for SSIM (*p* = 0.338). All deep learning methods significantly outperformed zero‐filled reconstruction (Figures 2 and 3).

KTMR demonstrated the greatest stability across AFs, with only a 4.5% decrease in PSNR from AF = 3 to AF = 10, compared to KIKI‐net (12.8%), ReconFormer (13.5%), and MR‐IPT (9.8%). Complete pairwise statistical comparisons are presented in Table S1.

Detailed boxplot comparisons illustrating the distribution of PSNR, SSIM, and NMSE values across all methods for AFs 3, 7, and 10 are provided in Figures S1–S9. Complete pairwise statistical comparisons are presented in Table S1.

To assess the clinical utility of KTMR reconstructions for quantitative lung function assessment, the agreement between VDP measurements derived from KTMR‐reconstructed images and those from semi‐automated analysis of fully sampled images across all AFs was evaluated.

**FIGURE 4:**
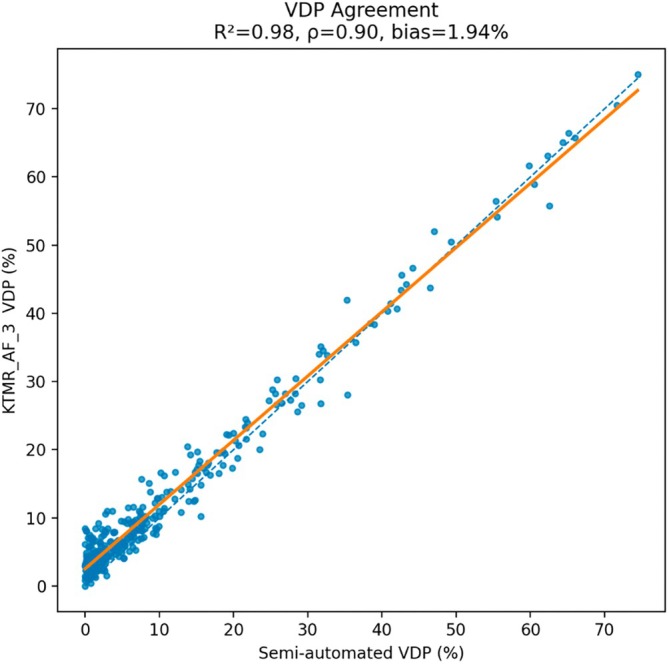
VDP correlation analysis at acceleration factor 3. Scatter plot showing agreement between KTMR‐reconstructed VDP at 3‐fold acceleration and reference VDP from fully sampled images. The orange solid line represents linear regression (*R* ^2^ = 0.975, slope = 0.942, intercept = 2.545, *p* < 0.001), and the dashed line represents the line of identity. Spearman correlation *ρ* = 0.905 (*p* < 0.001) with bias = 1.94%. Each data point represents a single 2D slice (*n* = 164 test slices). Tight clustering around the identity line demonstrates excellent agreement across the full VDP range, with the bias remaining below the clinically important 2% MCID threshold.

**FIGURE 5:**
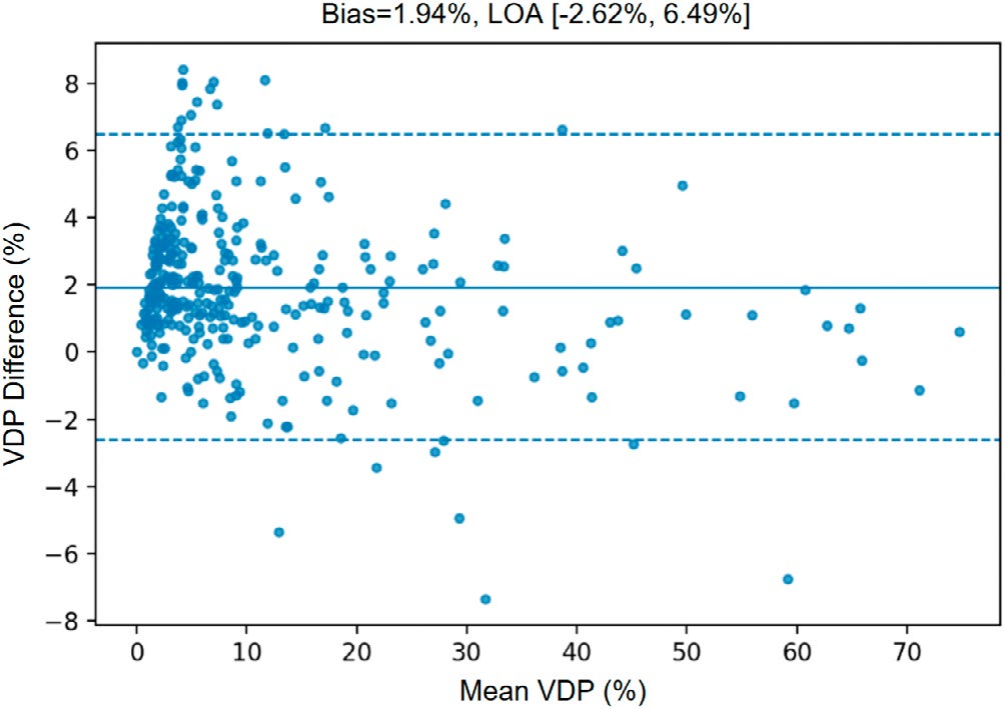
Bland–Altman agreement analysis at acceleration factor 3. Bland–Altman plot displaying the difference between KTMR_AF3 and reference VDP measurements as a function of the mean of both methods. The solid blue line represents the mean bias (1.94%), and dashed blue lines indicate 95% limits of agreement (LOA: −2.62% to 6.49%). Most differences cluster symmetrically around the mean bias with no apparent trend related to VDP magnitude, indicating consistent performance across disease severity. The bias remains below the 2% MCID, confirming clinical acceptability of 3‐fold acceleration for quantitative VDP assessment.

**FIGURE 6:**
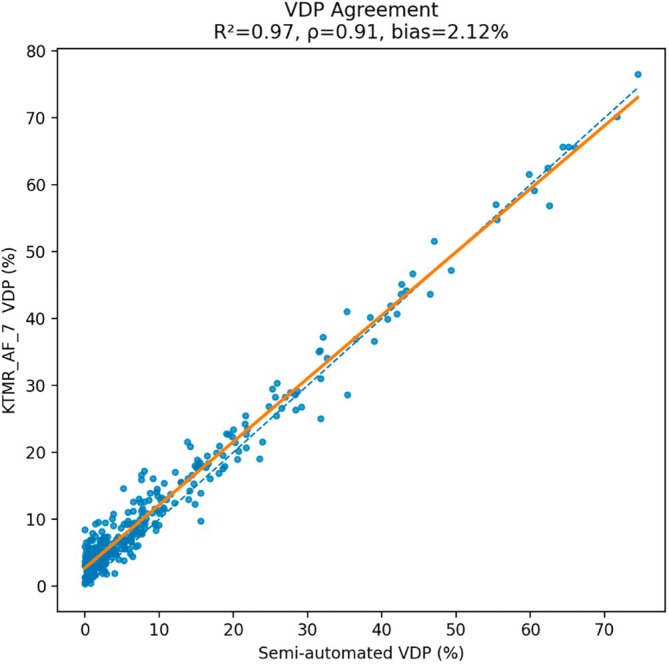
VDP correlation analysis at acceleration factor 7. Scatter plot showing KTMR_AF7 VDP agreement with semi‐automated measurements. Linear regression: *R* ^2^ = 0.973, slope = 0.945, intercept = 2.703 (*p* < 0.001); Spearman *ρ* = 0.905 (*p* < 0.001); bias = 2.12%. Despite 7‐fold acceleration (11.2‐fold total with partial echo), correlation remains nearly identical to AF3, with only minimal increase in scatter. The slight upward shift from the identity line reflects the small systematic bias of 2.12%, marginally above the 2% MCID but within acceptable clinical limits.

**FIGURE 7:**
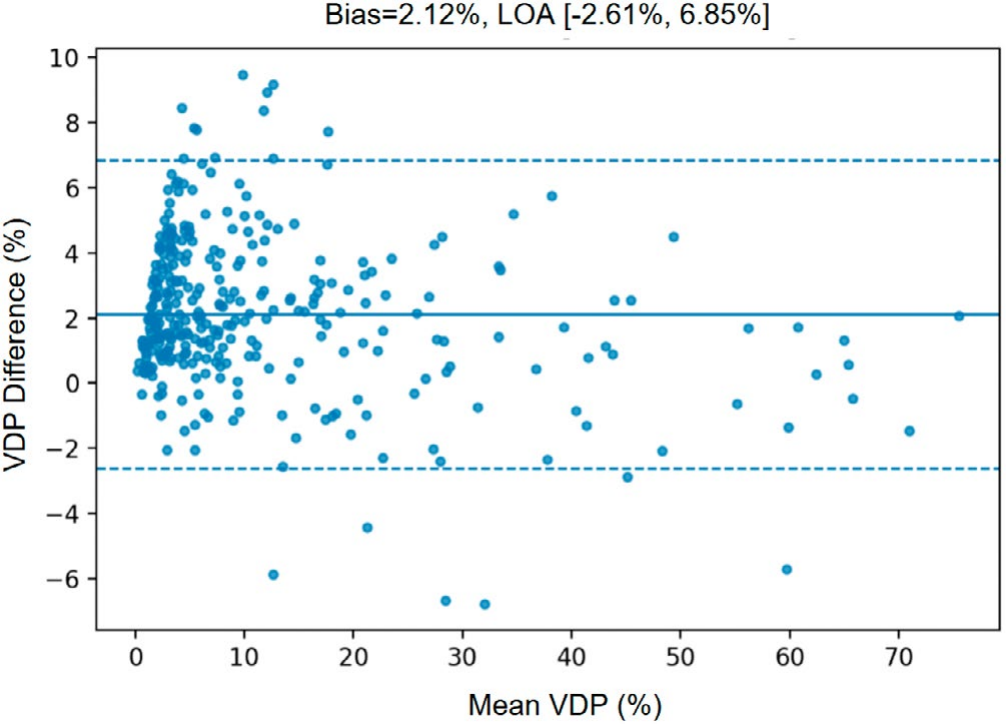
Bland–Altman agreement analysis at acceleration factor 7. Bland–Altman plot for AF7 showing mean bias = 2.12% with LOA [−2.61%, 6.85%]. The bias increased by only 0.18% compared to AF3, demonstrating KTMR's stability at higher acceleration. The width of the LOA (9.46%) remains narrow, indicating acceptable measurement precision. Scatter distribution remains symmetric without systematic trends, suggesting consistent quantitative performance across the VDP spectrum despite the aggressive undersampling.

**FIGURE 8:**
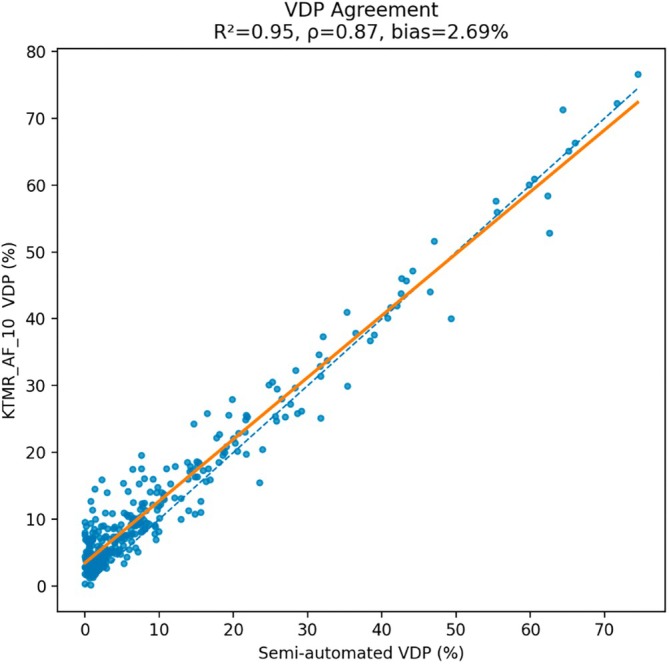
VDP correlation analysis at acceleration factor 10. Scatter plot showing KTMR_AF10 VDP versus reference VDP. Linear regression: *R* ^2^ = 0.953, slope = 0.926, intercept = 3.469 (*p* < 0.001); Spearman *ρ* = 0.871 (*p* < 0.001); bias = 2.69%. At 10‐fold acceleration (16‐fold total), correlation remains strong with *R* ^2^ exceeding 0.95, though increased scatter is evident compared to lower acceleration factors. The preserved correlation across the full VDP range indicates maintained relative accuracy despite the systematic bias exceeding the MCID, suggesting utility for screening and longitudinal monitoring applications.

**FIGURE 9:**
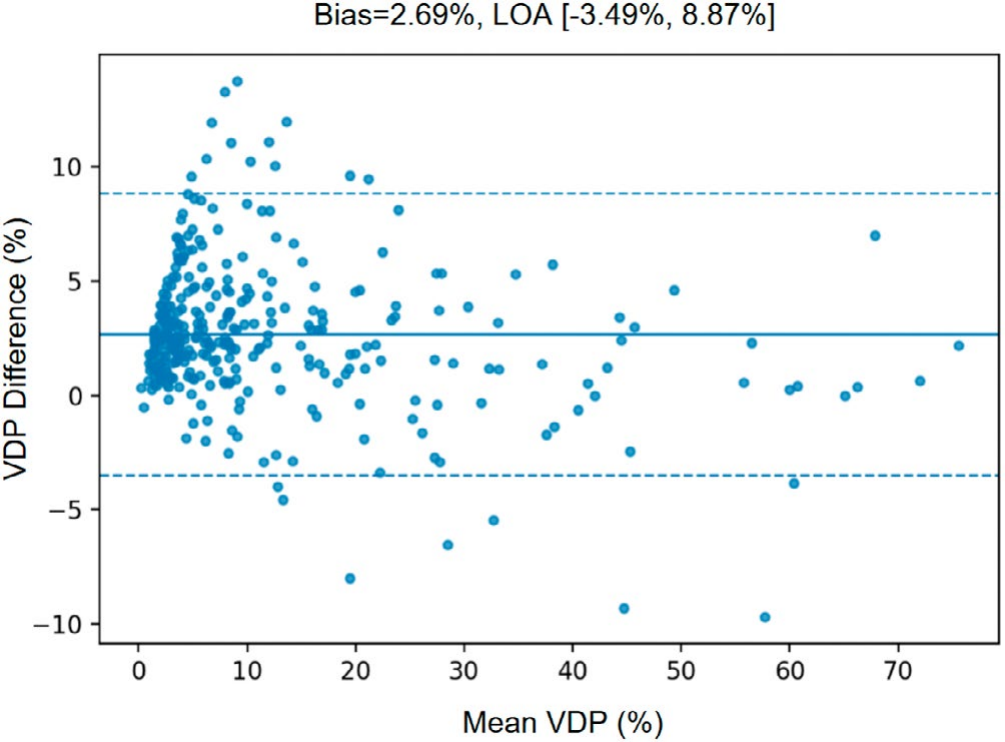
Bland–Altman agreement analysis at acceleration factor 10. Bland–Altman plot for AF10 showing mean bias = 2.69% with LOA [−3.50%, 8.87%]. The bias increased to 35% above the 2% MCID threshold, and the LOA width expanded to 12.37%, reflecting increased measurement variability at extreme acceleration. However, the majority of data points remain within reasonable limits, and no major outliers or systematic trends are evident. While absolute quantitative accuracy is reduced compared to AF3 and AF7, the symmetric distribution and absence of proportional bias suggest that 10‐fold acceleration remains viable for specific clinical scenarios prioritizing rapid acquisition over precise quantification.

Figures 4, 5, 6, 7, 8, 9 present correlation and agreement analyses across all three AFs. At AF3, linear regression analysis demonstrated a significant correlation between KTMR VDP and reference VDP (*R* ^2^ = 0.975, slope = 0.942, Spearman *ρ* = 0.905), with Bland–Altman analysis revealing a mean bias of 1.94% and 95% limits of agreement (LOA) of [−2.62%, 6.49%]. The mean bias remained below the established 2% MCID, indicating that KTMR at 3‐fold acceleration maintains clinically acceptable quantitative accuracy for VDP measurements.

At AF7, KTMR continued to show excellent correlation (*R* ^2^ = 0.973, slope = 0.945, *p* < 0.001; Spearman *ρ* = 0.905), with a Bland–Altman bias of 2.12% and LOA of [−2.61%, 6.85%]. The minimal increase in bias (1.94% → 2.12%) and negligible degradation in *R* ^2^ (0.975 → 0.973) demonstrate KTMR's remarkable stability at 7‐fold acceleration. While the bias exceeds the 2% MCID threshold by 0.12%, it remains within an acceptable clinical range, and the maintained Spearman correlation suggests consistent rank‐order preservation of VDP measurements across the full range of ventilation defect severity.

At the highest acceleration tested (AF10), KTMR maintained strong agreement (*R* ^2^ = 0.953, slope = 0.926, Spearman *ρ* = 0.871) despite the challenging undersampling conditions representing effective 16‐fold total acceleration when combined with the existing partial echo factor. The Bland–Altman bias increased to 2.69% with LOA of [−3.50%, 8.87%], exceeding the 2% MCID by approximately 35%. However, the preserved correlation coefficients indicate that relative VDP measurements remain reliable, suggesting that 10‐fold acceleration may be clinically viable for applications prioritizing speed over absolute quantitative precision.

For context, PSNR is measured on a logarithmic scale where each 3 dB increase corresponds to approximately halving the reconstruction error. Thus, KTMR's 7.5 dB advantage over zero‐filled reconstruction at AF = 3 (38.11 vs. 28.65 dB) represents roughly a five‐fold reduction in reconstruction error, while its 5.6 dB advantage over MR‐IPT at AF = 10 (36.41 vs. 28.84 dB) reflects approximately a four‐fold error reduction. Reconstructions above 30 dB are generally considered good quality, with values above 35 dB indicating excellent fidelity to the reference image.

## 4 Discussion

This study provides a systematic benchmark of transformer‐based and hybrid CNN–transformer architectures for HP ^129^Xe MRI reconstruction, demonstrating that the underlying architectural paradigm and the incorporation of physics‐informed constraints have significant impact on reconstruction fidelity, particularly at high AFs.

### 4.1 Hybrid Architecture Superiority

The performance of KTMR across all tested conditions can be understood through the complementary computational principles it integrates. By combining convolutional operations for local feature extraction with Swin Transformer blocks for capturing long‐range spatial dependencies, and anchoring both with explicit k‐space data consistency constraints, KTMR addresses the multiscale spatial structure of ventilation patterns in HP ^129^Xe MRI. This architectural synergy is consistent with emerging consensus in the MRI reconstruction literature that hybrid CNN‐transformer frameworks can outperform either paradigm in isolation, particularly at high AFs [12, 42]. The relative robustness of KTMR across AFs is particularly relevant to HP ^129^Xe imaging, where the finite and rapidly decaying polarization of the hyperpolarized gas places strict constraints on acquisition efficiency and necessitates high undersampling ratios [5]. The k‐space data consistency layer, which enforces that reconstructed k‐space values match acquired measurements at sampled locations, preserves the Fourier encoding relationship between k‐space and image space and constrains the network from generating physically inconsistent features in unsampled regions [38].

Ablation studies in the original KTMR framework confirmed the positive contribution of this layer across diverse imaging scenarios [42], and the present findings suggest this holds in the context of HP gas MRI.

### 4.2 Pure CNN Approaches

The competitive performance of KIKI‐net relative to all pure transformer architectures warrants discussion in the context of prevailing assumptions about architectural superiority in medical imaging reconstruction. While ViTs have attracted considerable attention for their capacity to model global context through self‐attention mechanisms [27], several studies have demonstrated that well‐designed CNN architectures with appropriate domain‐specific inductive biases remain highly competitive, particularly when training data are limited or domain structure can be explicitly exploited [27, 37]. KIKI‐net's iterative cross‐domain framework, which alternates between k‐space and image‐domain processing with interleaved data consistency operations, provides an effective mechanism for preserving both high‐frequency structural detail and low‐frequency signal integrity [41]. This dual‐domain strategy appears to compensate for the absence of global attention mechanisms, suggesting that physics‐informed processing pipelines may be a more critical determinant of reconstruction quality than the choice of feature extraction backbone alone [12, 38].

### 4.3 Limitations of Pure Transformer Approaches

The comparatively modest performance of pure transformer architectures in this setting reflects known challenges in applying ViTs to medical image reconstruction tasks, particularly under limited training data conditions. Transformers lack the spatial locality bias inherent to convolutional operations, which has been shown to confer advantages for image‐level tasks where local spatial coherence is important [27, 37]. SwinMR's hierarchical windowed attention mechanism, while computationally efficient, may sacrifice sensitivity to the fine‐grained local features critical for accurate ventilation defect boundary reconstruction, a limitation acknowledged in its original validation on brain MRI [42]. MR‐IPT's prompt‐guided multi‐task framework, designed for generalization across multiple reconstruction scenarios, may prioritize flexibility at the expense of task‐specific optimization, a trade‐off that may become increasingly detrimental under extreme undersampling conditions [44]. ReconFormer's recurrent pyramid structure, the most reconstruction‐specific design among the pure transformer architectures evaluated, achieved the strongest performance within this group, consistent with prior findings that task‐oriented architectural adaptations can partially offset the limitations of pure attention‐based processing [45]. Across all three pure transformer methods, the absence of explicit k‐space data consistency, a feature shared by both of the top‐performing architectures, may represent the most consequential structural difference, consistent with prior work emphasizing the importance of k‐space domain learning for accelerated MRI [38].

### 4.4 Clinical Implications and Translational Impact

The VDP agreement analyses situate reconstruction performance within a clinically meaningful framework defined by the established 2% MCID for ^129^Xe VDP in asthma [26]. At 3‐fold acceleration, the mean bias remained below this threshold, indicating quantitative equivalence with fully sampled acquisitions for VDP‐based clinical assessment, consistent with prior demonstrations that deep learning reconstruction can preserve quantitative ventilation biomarkers in HP gas MRI [23, 25]. At 7‐fold acceleration, the marginal increase in bias beyond the MCID threshold, in the context of preserved correlation and narrow limits of agreement, suggests that this level of acceleration may represent a pragmatic operating point for most clinical applications. Systematic biases of this magnitude have been shown to be amenable to prospective calibration in longitudinal monitoring contexts, where measurement consistency is more critical than absolute accuracy [25]. At 10‐fold acceleration, representing approximately 16‐fold total acceleration when combined with the existing partial echo factor, the more substantial increase in bias is consistent with expectations for extreme undersampling and should be interpreted cautiously for absolute VDP quantification [23]. Nevertheless, the preserved rank‐order correlation indicates maintained relative accuracy, suggesting potential utility for screening and within‐cohort comparative applications where acquisition speed is prioritized [23, 25].

The observed pattern of VDP‐dependent bias, with greater overestimation at lower VDP values, likely reflects the fundamental difficulty of reconstructing subtle or sparse ventilation defects from heavily undersampled data, where smoothing effects disproportionately affect small defect regions with limited image contrast [25]. This behavior has practical implications for patient selection and clinical interpretation, particularly in populations with near‐normal ventilation. Prospective bias correction models calibrated to VDP magnitude represent a practical avenue for improving absolute quantitative accuracy in future implementations.

Beyond static ventilation imaging, the acceleration capabilities evaluated here have broader relevance for HP gas MRI applications where SNR and temporal constraints are jointly limiting, including dynamic gas exchange imaging [47], diffusion‐weighted morphometry [24], and ^19^F dynamic lung MRI [48, 49], where accelerated sampling makes time available for additional signal averaging. The potential to enable imaging with natural‐abundance xenon through improved reconstruction efficiency has been demonstrated in prior work [3, 25], and the present findings provide further support for the role of deep learning reconstruction in reducing the effective isotope cost of HP ^129^Xe MRI, an important consideration for broader clinical accessibility.

### 4.5 Limitations

While this study establishes some benchmarks for transformer‐based HP ^129^Xe MRI reconstruction, several limitations warrant consideration. The evaluation focused exclusively on static ventilation imaging, and future work should extend these findings to dynamic gas exchange imaging and diffusion‐weighted applications.

The single‐institution design, while enabling standardized acquisition protocols, may limit generalizability across different scanner platforms, polarization systems, and acquisition parameters. Multi‐center validation studies will be important to confirm the robustness of these methods across diverse clinical environments and patient populations.

The retrospective nature of the acceleration simulation, while computationally efficient and enabling controlled comparison across AFs, does not fully replicate the noise characteristics and acquisition artifacts that occur during prospective undersampled acquisition. Prospective validation studies with actual accelerated acquisition protocols will be necessary to confirm clinical translation potential.

The focus on 2D reconstruction, while consistent with current clinical HP ^129^Xe imaging protocols, may not fully exploit the potential advantages of transformer architectures for 3D volumetric reconstruction.

It should be noted that the iterative conversion between k‐space and image domains in KIKI‐net's processing pipeline does not explicitly model the non‐equilibrium signal evolution inherent to hyperpolarized gas acquisitions, where RF‐induced magnetization depletion causes signal variation across k‐space lines. However, because the retrospective undersampling was applied to previously acquired k‐space data in which these signal variations are already encoded, the data consistency operations enforce fidelity to the original measurements inclusive of these effects rather than introducing additional distortion.

This study was conducted at a single center, using a single vendor platform (GE Healthcare Discovery MR750), at a single field strength (3 T), with a single 3D GRE pulse sequence employing Cartesian sampling, a specific semi‐automated VDP quantification pipeline, and retrospective undersampling rather than prospective acceleration. While this design enabled standardized acquisition protocols and controlled comparisons across architectures, it may limit generalizability across different scanner vendors, field strengths, polarization systems, and clinical environments. The trained models are specific to the acquired matrix size (128 × 128), resolution, and k‐space sampling trajectory and would require retraining for substantially different acquisition strategies including 2D multi‐slice, spiral, 3D radial, or FLORET trajectories, as well as different spatial resolutions. The learned k‐space completion patterns are inherently tied to the Cartesian undersampling used here and would not generalize to non‐Cartesian trajectories. Similarly, the VDP quantification results are specific to the semi‐automated method employed and may differ with alternative quantification approaches.

## 5 Conclusion

This proof‐of‐concept study indicates that hybrid CNN–transformer architectures incorporating explicit k‐space data consistency constraints may provide reconstruction advantages over pure transformer and pure CNN approaches for accelerated HP ^129^Xe MRI in a single‐center retrospective setting. The findings suggest that dual‐domain processing, rather than architectural complexity alone, is a key determinant of reconstruction quality across the evaluated AFs. Prospective, multi‐center validation will be required to determine the generalizability of these results across different acquisition platforms, patient populations, and clinical workflows.

## Funding

This work was supported by the Natural Sciences and Engineering Research Council of Canada, R9245A04.

## Supporting information


**Figure S1:** NMSE comparison across all deep learning methods and zero‐filled reconstruction at acceleration factor 3 (AF3). Boxplots display the distribution of normalized mean squared error (NMSE) values, where lower values indicate superior reconstruction accuracy. KTMR (hybrid CNN‐transformer) and KIKI‐net (pure CNN) demonstrate significantly lower NMSE values compared to all other methods, with KIKI‐net showing the lowest median NMSE and tightest interquartile range. Pure transformer methods (MR‐IPT, ReconFormer, SwinMR) exhibit progressively higher NMSE values, while zero‐filled reconstruction shows the worst performance. Statistical significance brackets indicate *p*‐values from post hoc Dunn's test with Benjamini‐Hochberg correction following the Friedman test. Note that KTMR and KIKI‐net show non‐significant difference (ns) between each other but significant differences (*p* < 0.001) compared to all other methods, confirming the superior performance of hybrid and CNN approaches over pure transformer architectures.
**Figure S2:** PSNR comparison across all deep learning methods and zero‐filled reconstruction at acceleration factor 3 (AF3). Boxplots display the distribution of Peak Signal‐to‐Noise Ratio (PSNR) values in decibels (dB), where higher values indicate superior reconstruction fidelity. KTMR (hybrid CNN‐transformer) achieves the highest median PSNR (~38 dB), followed closely by KIKI‐net (pure CNN) (~37 dB), with no statistically significant difference between these top two performers (ns). Pure transformer methods demonstrate progressively declining performance: MR‐IPT (~32 dB), ReconFormer (~34 dB), and SwinMR (~30 dB), all significantly lower than the hybrid and CNN approaches. Zero‐filled reconstruction exhibits the poorest performance (~28 dB). Statistical significance brackets indicate *p*‐values from post hoc Dunn's test with Benjamini‐Hochberg correction following the Friedman test. The results confirm that hybrid CNN‐transformer and pure CNN architectures significantly outperform pure transformer approaches (*p* < 0.001 for all comparisons), establishing the superiority of hybrid and CNN‐based reconstruction methods for HP ^129^Xe MRI at moderate acceleration factors.
**Figure S3:** SSIM comparison across all deep learning methods and zero‐filled reconstruction at acceleration factor 3 (AF3). Boxplots display the distribution of structural similarity index measure (SSIM) values, where higher values (closer to 1.0) indicate superior perceptual reconstruction quality and structural preservation. KTMR (hybrid CNN‐transformer) achieves the highest median SSIM (~0.95), followed closely by KIKI‐net (pure CNN) (~0.93), both demonstrating excellent structural fidelity. Pure transformer methods show progressively declining performance: ReconFormer (~0.86), MR‐IPT (~0.83), and SwinMR (~0.79), all significantly lower than the hybrid and CNN approaches. Zero‐filled reconstruction exhibits the poorest structural similarity (~0.76). Statistical significance brackets indicate *p*‐values from post hoc Dunn's test with Benjamini‐Hochberg correction following the Friedman test. All pairwise comparisons demonstrate significant differences (*p* < 0.001), confirming the hierarchical performance ranking: KTMR ≥ KIKI‐net >> ReconFormer > MR‐IPT > SwinMR > Zero‐filling. The results emphasize that hybrid CNN‐transformer and pure CNN architectures achieve superior structural preservation compared to pure transformer approaches in HP ^129^Xe MRI reconstruction.
**Figure S4:** NMSE comparison across all deep learning methods and zero‐filled reconstruction at acceleration factor 7 (AF7). Boxplots display the distribution of normalized mean squared error (NMSE) values, where lower values indicate superior reconstruction accuracy. At this higher acceleration factor, KTMR (hybrid CNN‐transformer) and KIKI‐net (pure CNN) maintain their superior performance with the lowest median NMSE values (~0.015) and show no statistically significant difference between each other (ns). Pure transformer methods demonstrate increased reconstruction errors compared to AF3: MR‐IPT (~0.035), ReconFormer (~0.025), and SwinMR (~0.035), with some methods showing non‐significant differences among themselves (ns) but all significantly worse than the top performers. Zero‐filled reconstruction exhibits substantially degraded performance (~0.065) at this acceleration level. Statistical significance brackets indicate *p*‐values from post hoc Dunn's test with Benjamini‐Hochberg correction following the Friedman test. The results demonstrate that hybrid CNN‐transformer and pure CNN architectures maintain robust reconstruction accuracy even at aggressive acceleration factors, while pure transformer approaches show notable performance degradation, emphasizing the importance of CNN components for reliable HP ^129^Xe MRI reconstruction.
**Figure S5:** PSNR comparison across all deep learning methods and zero‐filled reconstruction at acceleration factor 7 (AF7). Boxplots display the distribution of peak signal‐to‐noise ratio (PSNR) values in decibels (dB), where higher values indicate superior reconstruction fidelity. At this higher acceleration factor, KTMR (hybrid CNN‐transformer) maintains the highest median PSNR (~37 dB), demonstrating exceptional robustness to increased undersampling. KIKI‐net (pure CNN) achieves the second‐best performance (~34 dB), with both top performers showing significant superiority over all other methods (*p* < 0.001). Among pure transformer methods, ReconFormer (~32 dB) and MR‐IPT (~31 dB) show non‐significant differences (ns) between each other but both significantly outperform SwinMR (~30 dB). Zero‐filled reconstruction exhibits the poorest performance (~28 dB). Statistical significance brackets indicate *p*‐values from post hoc Dunn's test with Benjamini‐Hochberg correction following the Friedman test. Compared to AF3 results, all methods show expected performance degradation at this aggressive acceleration level, but KTMR maintains superior stability with minimal quality loss, while pure transformer approaches demonstrate more pronounced degradation, reinforcing the advantage of hybrid CNN‐transformer architectures for challenging reconstruction scenarios.
**Figure S6:** SSIM comparison across all deep learning methods and zero‐filled reconstruction at acceleration factor 7 (AF7). Boxplots display the distribution of structural similarity index measure (SSIM) values, where higher values (closer to 1.0) indicate superior perceptual reconstruction quality and structural preservation. At this aggressive acceleration level, KTMR (hybrid CNN‐transformer) maintains the highest median SSIM (~0.90), demonstrating exceptional robustness in preserving structural fidelity despite increased undersampling. KIKI‐net (pure CNN) achieves the second‐best performance (~0.85), with both top performers significantly outperforming all other methods (*p* < 0.001). Among pure transformer methods, ReconFormer (~0.82) and MR‐IPT (~0.79) show non‐significant differences (ns) between each other, while SwinMR exhibits further degradation (~0.76). Zero‐filled reconstruction shows the poorest structural similarity (~0.71). Statistical significance brackets indicate *p*‐values from post hoc Dunn's test with Benjamini‐Hochberg correction following the Friedman test. Compared to AF3, all methods demonstrate expected structural quality degradation at this higher acceleration factor, but KTMR and KIKI‐net maintain superior structural preservation capabilities, emphasizing the critical importance of CNN components for maintaining perceptual quality in challenging HP ^129^Xe MRI reconstruction scenarios.
**Figure S7:** NMSE comparison across all deep learning methods and zero‐filled reconstruction at acceleration factor 10 (AF10). Boxplots display the distribution of normalized mean squared error (NMSE) values, where lower values indicate superior reconstruction accuracy. At this most aggressive acceleration level, KTMR (hybrid CNN‐transformer) and KIKI‐net (pure CNN) continue to demonstrate exceptional reconstruction fidelity with the lowest median NMSE values (~0.020), significantly outperforming all other methods (*p* < 0.001). Pure transformer methods exhibit substantial error increases at this extreme acceleration: MR‐IPT (~0.055) and ReconFormer (~0.048) show non‐significant differences (ns) between each other, while SwinMR maintains similar error levels (~0.040) to lower acceleration factors. Zero‐filled reconstruction demonstrates severely degraded performance (~0.10) at this acceleration level. Statistical significance brackets indicate *p*‐values from post hoc Dunn's test with Benjamini‐Hochberg correction following the Friedman test. The results emphasize the remarkable robustness of hybrid CNN‐transformer and pure CNN architectures even under extreme undersampling conditions (10% of original data), while pure transformer approaches struggle significantly, demonstrating the critical importance of CNN components for reliable HP ^129^Xe MRI reconstruction at clinically relevant acceleration factors.
**Figure S8:** PSNR comparison across all deep learning methods and zero‐filled reconstruction at acceleration factor 10 (AF10). Boxplots display the distribution of peak signal‐to‐noise ratio (PSNR) values in decibels (dB), where higher values indicate superior reconstruction fidelity. At this most aggressive acceleration level (only 10% of original data), KTMR (hybrid CNN‐transformer) demonstrates exceptional robustness with the highest median PSNR (~36 dB), maintaining clinically viable reconstruction quality even under extreme undersampling. KIKI‐net (pure CNN) achieves solid second‐place performance (~32 dB), significantly outperforming all pure transformer methods (*p* < 0.001). Pure transformer approaches show substantial degradation: SwinMR (~30 dB), ReconFormer (~30 dB), and MR‐IPT (~29 dB) cluster together with relatively similar performance levels, all significantly worse than the hybrid and CNN methods. Zero‐filled reconstruction exhibits severely compromised quality (~26 dB). Statistical significance brackets indicate *p*‐values from post hoc Dunn's test with Benjamini‐Hochberg correction following the Friedman test. The results demonstrate that KTMR's hybrid architecture maintains remarkable stability across all acceleration factors, with only 4.5% PSNR degradation from AF3 to AF10, while pure transformer methods suffer more dramatic quality losses, emphasizing the critical importance of CNN components for extreme acceleration scenarios in HP ^129^Xe MRI reconstruction.
**Figure S9:** SSIM comparison across all deep learning methods and zero‐filled reconstruction at acceleration factor 10 (AF10). Boxplots display the distribution of structural similarity index measure (SSIM) values, where higher values (closer to 1.0) indicate superior perceptual reconstruction quality and structural preservation. At this most extreme acceleration level (only 10% of original data), KTMR (hybrid CNN‐transformer) maintains exceptional structural fidelity with the highest median SSIM (~0.88), demonstrating remarkable robustness under severe undersampling conditions. KIKI‐net (pure CNN) achieves solid second‐place performance (~0.82), with both top performers significantly outperforming all other methods (*p* < 0.001). Among pure transformer methods, MR‐IPT (~0.75) and ReconFormer (~0.77) show non‐significant differences (ns) between each other, while SwinMR (~0.76) performs similarly, with some non‐significant differences (ns) noted between transformer approaches. Zero‐filled reconstruction exhibits severely compromised structural similarity (~0.65). Statistical significance brackets indicate *p*‐values from post hoc Dunn's test with Benjamini‐Hochberg correction following the Friedman test. These results demonstrate that KTMR's hybrid architecture maintains superior structural preservation capabilities even under the most challenging reconstruction scenarios, while pure transformer methods show substantial structural quality degradation, confirming the critical importance of CNN components for maintaining perceptual fidelity in extreme acceleration HP ^129^Xe MRI reconstruction.
**Table S1:** Pairwise statistical comparisons of reconstruction performance across deep learning architectures.
