# Supplementary material for: Benchmarking Hybrid CNN‐Transformer Versus Pure Transformer Architectures for Accelerated Hyperpolarized 129Xe MRI Reconstruction
Source: J Magn Reson Imaging. 2026 Mar 27;64(1):266–80. doi: 10.1002/jmri.70314 (PMC13254042; doi:10.1002/jmri.70314)
Supplement: Supplementary file 1 — Figure S1: NMSE comparison across all deep learning methods and zero‐filled reconstruction at acceleration factor 3 (AF3). Boxplots display the distribution of normalized mean squared error (NMSE) values, where lower values indicate superior reconstruction accuracy. KTMR (hybrid CNN‐transformer) and KIKI‐net (pure CNN) demonstrate significantly lower NMSE values compared to all other methods, with KIKI‐net showing the lowest median NMSE and tightest interquartile range. Pure transformer methods (MR‐IPT, ReconFormer, SwinMR) exhibit progressively higher NMSE values, while zero‐filled reconstruction shows the worst performance. Statistical significance brackets indicate p‐values from post hoc Dunn's test with Benjamini‐Hochberg correction following the Friedman test. Note that KTMR and KIKI‐net show non‐significant difference (ns) between each other but significant differences (p < 0.001) compared to all other methods, confirming the superior performance of hybrid and CNN approaches over pure transformer architectures. Figure S2: PSNR comparison across all deep learning methods and zero‐filled reconstruction at acceleration factor 3 (AF3). Boxplots display the distribution of Peak Signal‐to‐Noise Ratio (PSNR) values in decibels (dB), where higher values indicate superior reconstruction fidelity. KTMR (hybrid CNN‐transformer) achieves the highest median PSNR (~38 dB), followed closely by KIKI‐net (pure CNN) (~37 dB), with no statistically significant difference between these top two performers (ns). Pure transformer methods demonstrate progressively declining performance: MR‐IPT (~32 dB), ReconFormer (~34 dB), and SwinMR (~30 dB), all significantly lower than the hybrid and CNN approaches. Zero‐filled reconstruction exhibits the poorest performance (~28 dB). Statistical significance brackets indicate p‐values from post hoc Dunn's test with Benjamini‐Hochberg correction following the Friedman test. The results confirm that hybrid CNN‐transformer and pure CNN architectur [file JMRI-64-266-s001.docx]

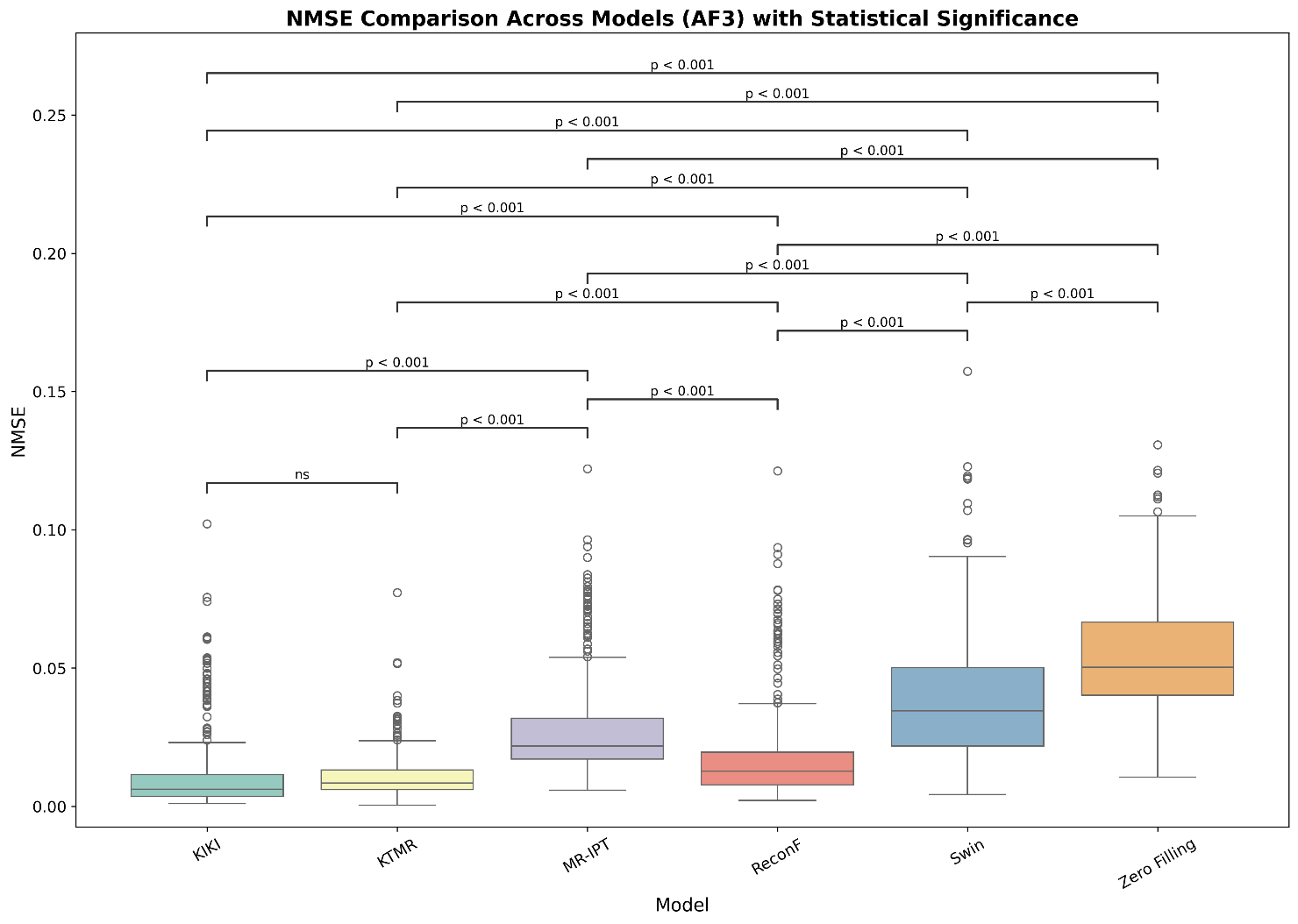


**Figure S1. NMSE comparison across all deep learning methods and zero-filled reconstruction at acceleration factor 3 (AF3).** Boxplots display the distribution of Normalized Mean Squared Error (NMSE) values, where lower values indicate superior reconstruction accuracy. KTMR (hybrid CNN-transformer) and KIKI-net (pure CNN) demonstrate significantly lower NMSE values compared to all other methods, with KIKI-net showing the lowest median NMSE and tightest interquartile range. Pure transformer methods (MR-IPT, ReconFormer, SwinMR) exhibit progressively higher NMSE values, while zero-filled reconstruction shows the worst performance. Statistical significance brackets indicate p-values from post-hoc Dunn's test with Benjamini-Hochberg correction following the Friedman test. Note that KTMR and KIKI-net show non-significant difference (ns) between each other but significant differences (p < 0.001) compared to all other methods, confirming the superior performance of hybrid and CNN approaches over pure transformer architectures.


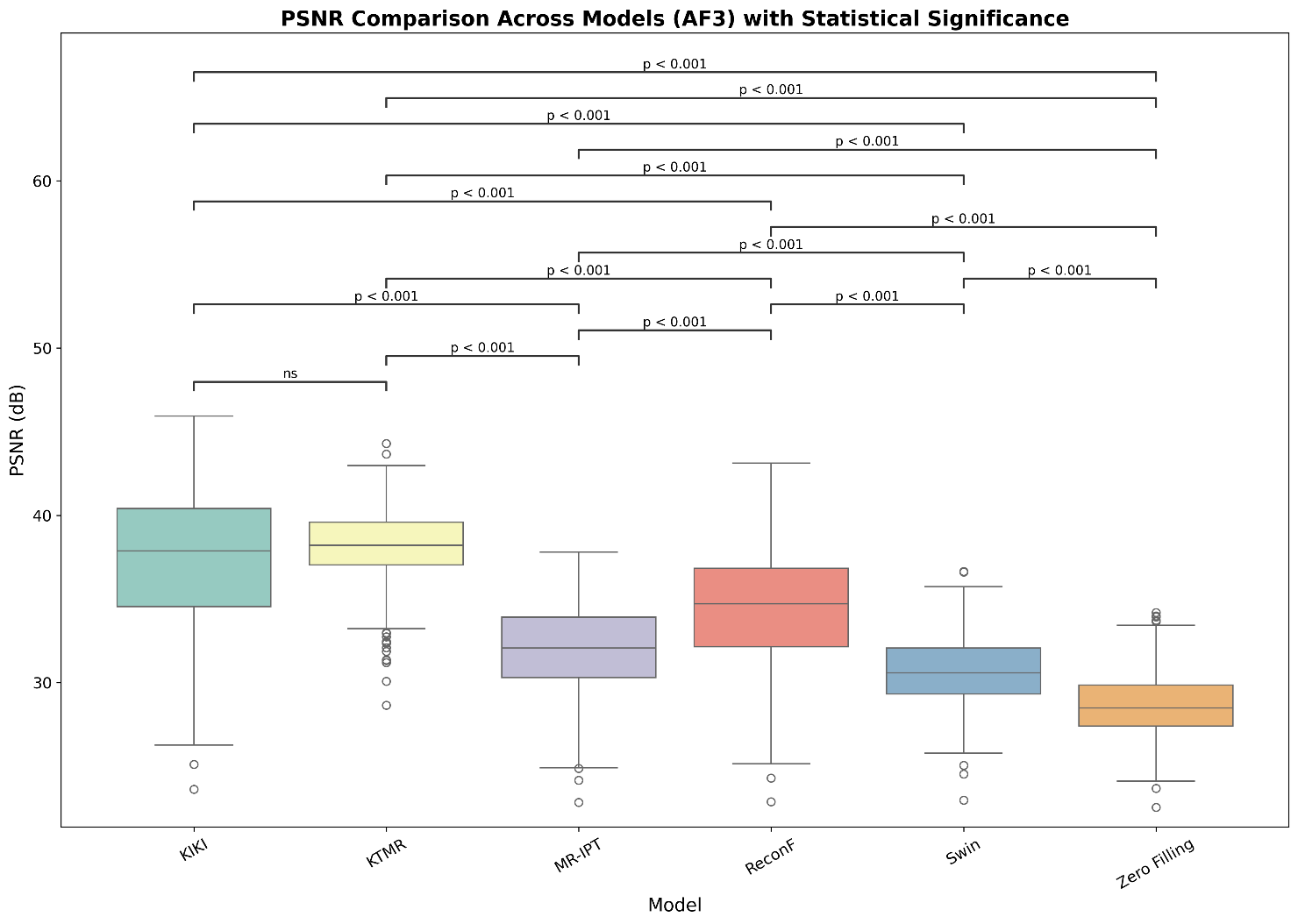


**Figure S2. PSNR comparison across all deep learning methods and zero-filled reconstruction at acceleration factor 3 (AF3).** Boxplots display the distribution of Peak Signal-to-Noise Ratio (PSNR) values in decibels (dB), where higher values indicate superior reconstruction fidelity. KTMR (hybrid CNN-transformer) achieves the highest median PSNR (~38 dB), followed closely by KIKI-net (pure CNN) (~37 dB), with no statistically significant difference between these top two performers (ns). Pure transformer methods demonstrate progressively declining performance: MR-IPT (~32 dB), ReconFormer (~34 dB), and SwinMR (~30 dB), all significantly lower than the hybrid and CNN approaches. Zero-filled reconstruction exhibits the poorest performance (~28 dB). Statistical significance brackets indicate p-values from post-hoc Dunn's test with Benjamini-Hochberg correction following the Friedman test. The results confirm that hybrid CNN-transformer and pure CNN architectures significantly outperform pure transformer approaches (p < 0.001 for all comparisons), establishing the superiority of hybrid and CNN-based reconstruction methods for HP ¹²⁹Xe MRI at moderate acceleration factors.


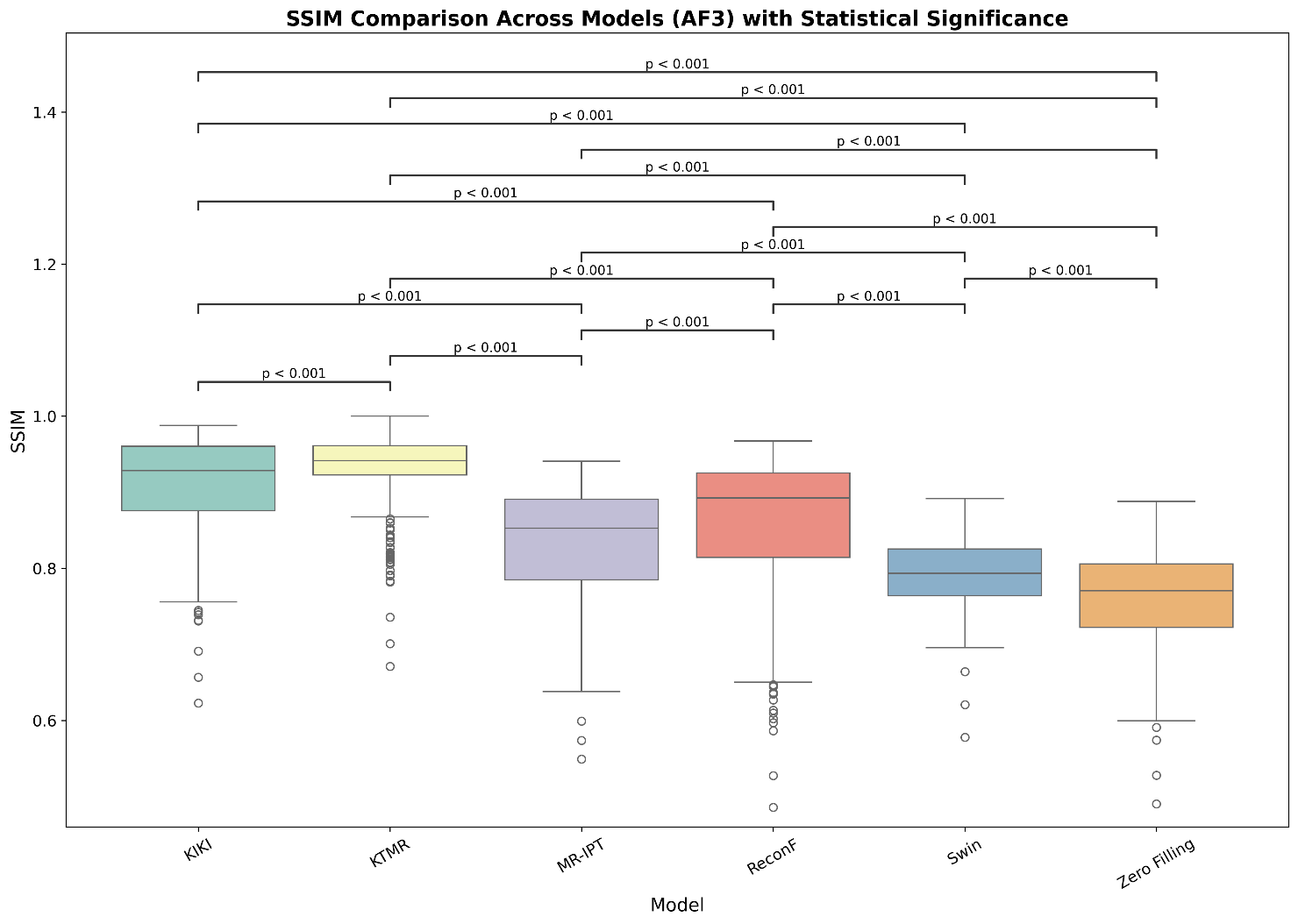


**Figure S3. SSIM comparison across all deep learning methods and zero-filled reconstruction at acceleration factor 3 (AF3).** Boxplots display the distribution of Structural Similarity Index Measure (SSIM) values, where higher values (closer to 1.0) indicate superior perceptual reconstruction quality and structural preservation. KTMR (hybrid CNN-transformer) achieves the highest median SSIM (~0.95), followed closely by KIKI-net (pure CNN) (~0.93), both demonstrating excellent structural fidelity. Pure transformer methods show progressively declining performance: ReconFormer (~0.86), MR-IPT (~0.83), and SwinMR (~0.79), all significantly lower than the hybrid and CNN approaches. Zero-filled reconstruction exhibits the poorest structural similarity (~0.76). Statistical significance brackets indicate p-values from post-hoc Dunn's test with Benjamini-Hochberg correction following the Friedman test. All pairwise comparisons demonstrate significant differences (p < 0.001), confirming the hierarchical performance ranking: KTMR ≥ KIKI-net >> ReconFormer > MR-IPT > SwinMR > Zero-filling. The results emphasize that hybrid CNN-transformer and pure CNN architectures achieve superior structural preservation compared to pure transformer approaches in HP ¹²⁹Xe MRI reconstruction.


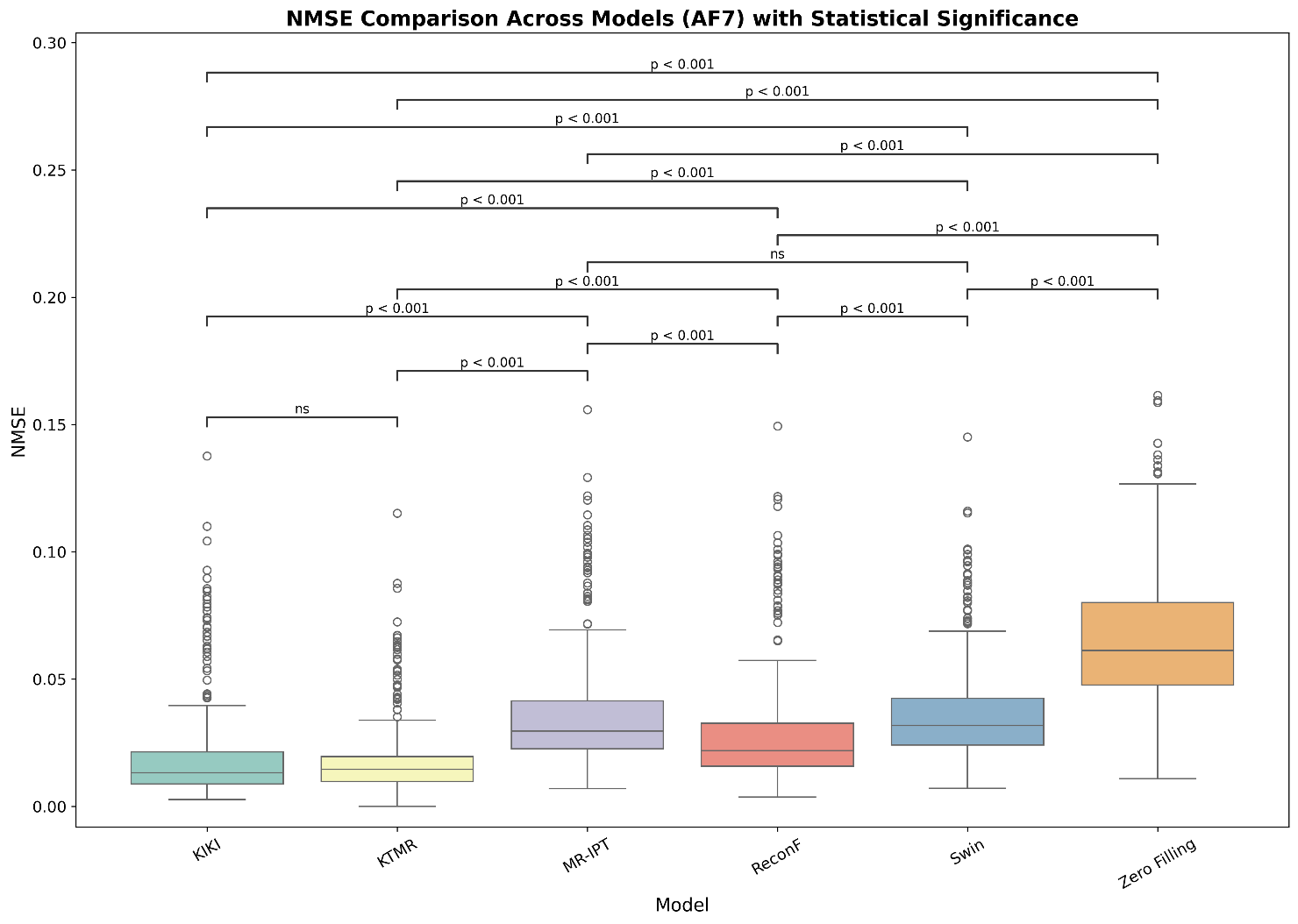


**Figure S4. NMSE comparison across all deep learning methods and zero-filled reconstruction at acceleration factor 7 (AF7).** Boxplots display the distribution of Normalized Mean Squared Error (NMSE) values, where lower values indicate superior reconstruction accuracy. At this higher acceleration factor, KTMR (hybrid CNN-transformer) and KIKI-net (pure CNN) maintain their superior performance with the lowest median NMSE values (~0.015) and show no statistically significant difference between each other (ns). Pure transformer methods demonstrate increased reconstruction errors compared to AF3: MR-IPT (~0.035), ReconFormer (~0.025), and SwinMR (~0.035), with some methods showing non-significant differences among themselves (ns) but all significantly worse than the top performers. Zero-filled reconstruction exhibits substantially degraded performance (~0.065) at this acceleration level. Statistical significance brackets indicate p-values from post-hoc Dunn's test with Benjamini-Hochberg correction following the Friedman test. The results demonstrate that hybrid CNN-transformer and pure CNN architectures maintain robust reconstruction accuracy even at aggressive acceleration factors, while pure transformer approaches show notable performance degradation, emphasizing the importance of CNN components for reliable HP ¹²⁹Xe MRI reconstruction.


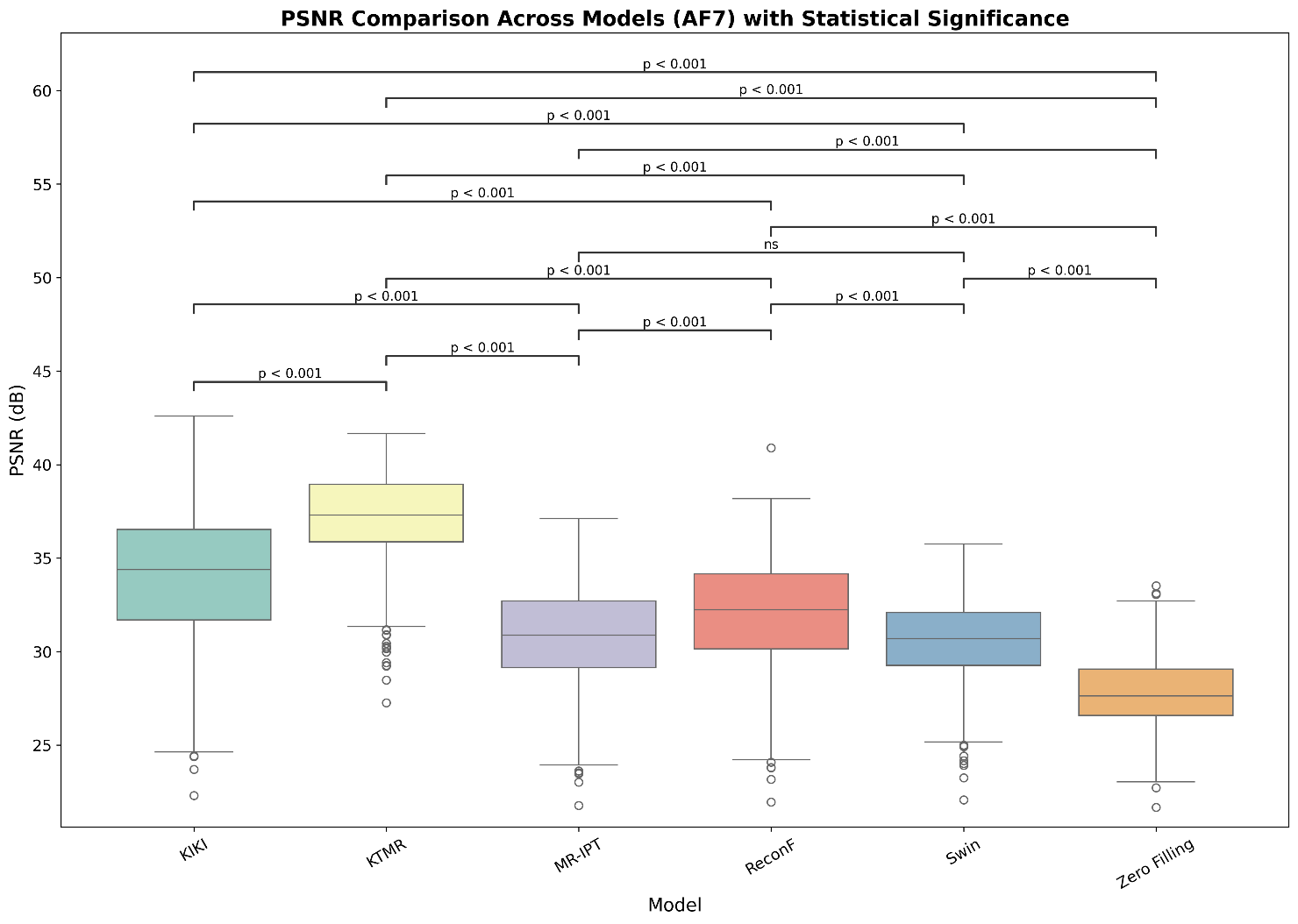


**Figure S5. PSNR comparison across all deep learning methods and zero-filled reconstruction at acceleration factor 7 (AF7).** Boxplots display the distribution of Peak Signal-to-Noise Ratio (PSNR) values in decibels (dB), where higher values indicate superior reconstruction fidelity. At this higher acceleration factor, KTMR (hybrid CNN-transformer) maintains the highest median PSNR (~37 dB), demonstrating exceptional robustness to increased undersampling. KIKI-net (pure CNN) achieves the second-best performance (~34 dB), with both top performers showing significant superiority over all other methods (p < 0.001). Among pure transformer methods, ReconFormer (~32 dB) and MR-IPT (~31 dB) show non-significant differences (ns) between each other but both significantly outperform SwinMR (~30 dB). Zero-filled reconstruction exhibits the poorest performance (~28 dB). Statistical significance brackets indicate p-values from post-hoc Dunn's test with Benjamini-Hochberg correction following the Friedman test. Compared to AF3 results, all methods show expected performance degradation at this aggressive acceleration level, but KTMR maintains superior stability with minimal quality loss, while pure transformer approaches demonstrate more pronounced degradation, reinforcing the advantage of hybrid CNN-transformer architectures for challenging reconstruction scenarios.


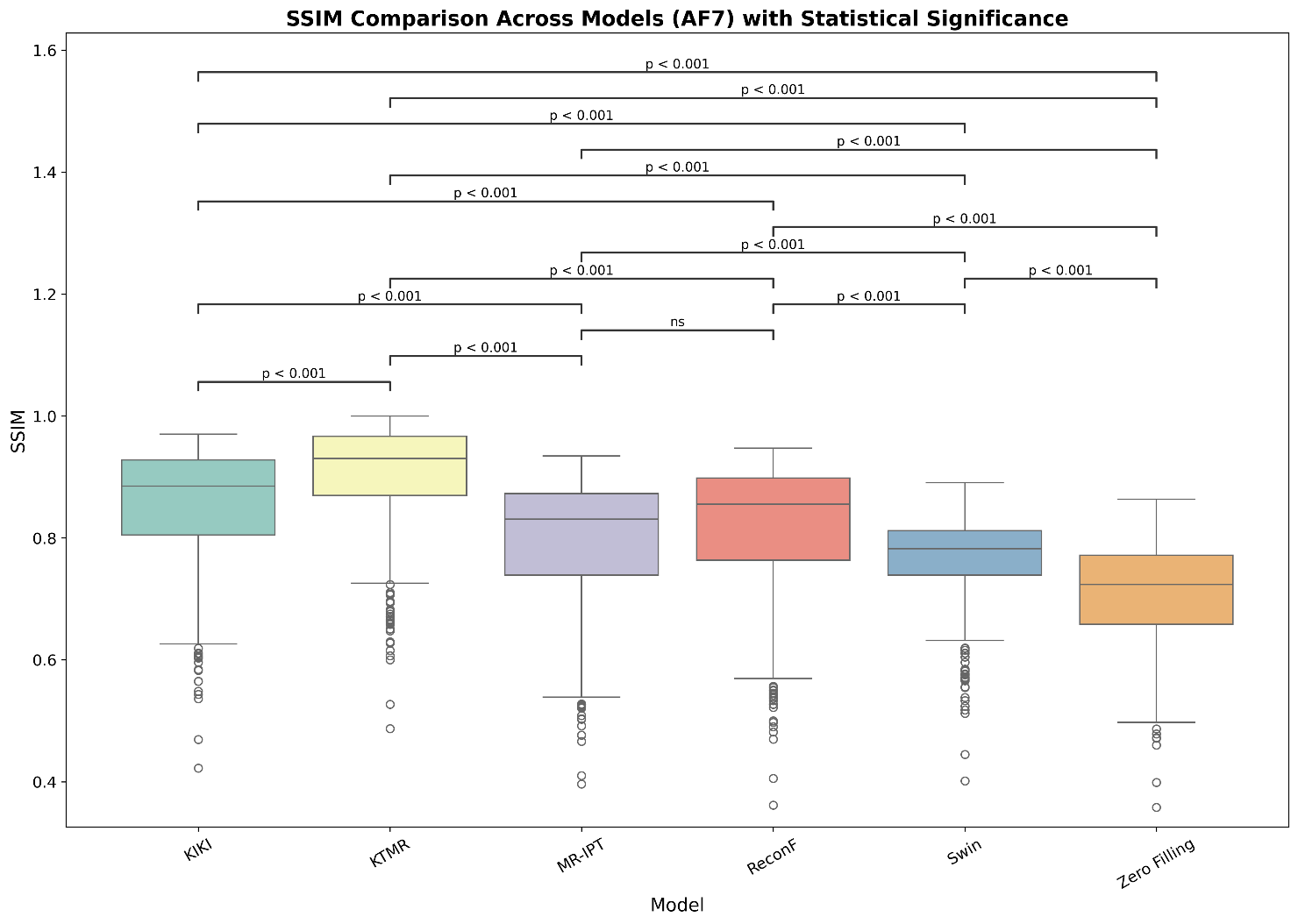


**Figure S6. SSIM comparison across all deep learning methods and zero-filled reconstruction at acceleration factor 7 (AF7).** Boxplots display the distribution of Structural Similarity Index Measure (SSIM) values, where higher values (closer to 1.0) indicate superior perceptual reconstruction quality and structural preservation. At this aggressive acceleration level, KTMR (hybrid CNN-transformer) maintains the highest median SSIM (~0.90), demonstrating exceptional robustness in preserving structural fidelity despite increased undersampling. KIKI-net (pure CNN) achieves the second-best performance (~0.85), with both top performers significantly outperforming all other methods (p < 0.001). Among pure transformer methods, ReconFormer (~0.82) and MR-IPT (~0.79) show non-significant differences (ns) between each other, while SwinMR exhibits further degradation (~0.76). Zero-filled reconstruction shows the poorest structural similarity (~0.71). Statistical significance brackets indicate p-values from post-hoc Dunn's test with Benjamini-Hochberg correction following the Friedman test. Compared to AF3, all methods demonstrate expected structural quality degradation at this higher acceleration factor, but KTMR and KIKI-net maintain superior structural preservation capabilities, emphasizing the critical importance of CNN components for maintaining perceptual quality in challenging HP ¹²⁹Xe MRI reconstruction scenarios.


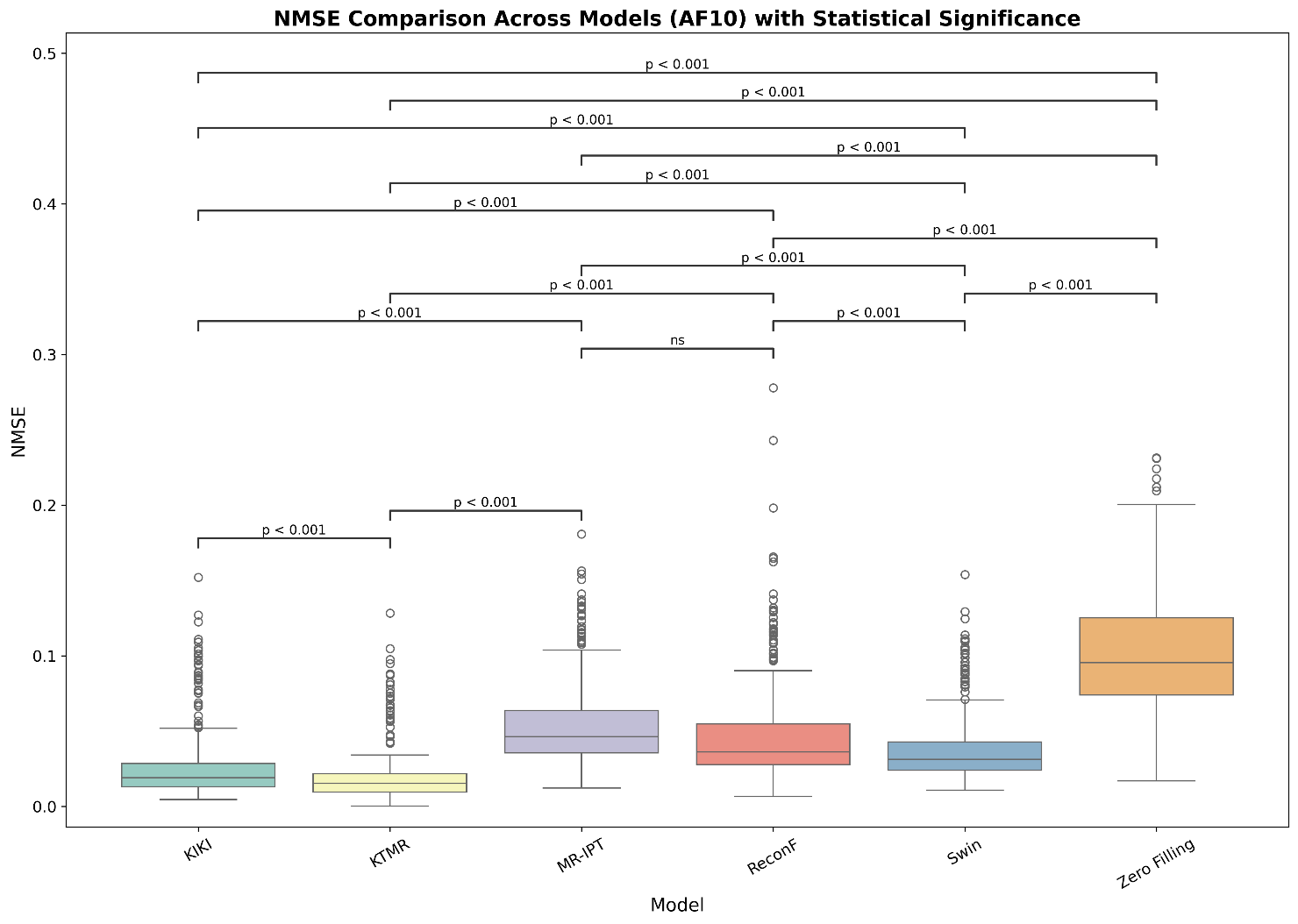


**Figure S7. NMSE comparison across all deep learning methods and zero-filled reconstruction at acceleration factor 10 (AF10).** Boxplots display the distribution of Normalized Mean Squared Error (NMSE) values, where lower values indicate superior reconstruction accuracy. At this most aggressive acceleration level, KTMR (hybrid CNN-transformer) and KIKI-net (pure CNN) continue to demonstrate exceptional reconstruction fidelity with the lowest median NMSE values (~0.020), significantly outperforming all other methods (p < 0.001). Pure transformer methods exhibit substantial error increases at this extreme acceleration: MR-IPT (~0.055) and ReconFormer (~0.048) show non-significant differences (ns) between each other, while SwinMR maintains similar error levels (~0.040) to lower acceleration factors. Zero-filled reconstruction demonstrates severely degraded performance (~0.10) at this acceleration level. Statistical significance brackets indicate p-values from post-hoc Dunn's test with Benjamini-Hochberg correction following the Friedman test. The results emphasize the remarkable robustness of hybrid CNN-transformer and pure CNN architectures even under extreme undersampling conditions (10% of original data), while pure transformer approaches struggle significantly, demonstrating the critical importance of CNN components for reliable HP ¹²⁹Xe MRI reconstruction at clinically relevant acceleration factors.


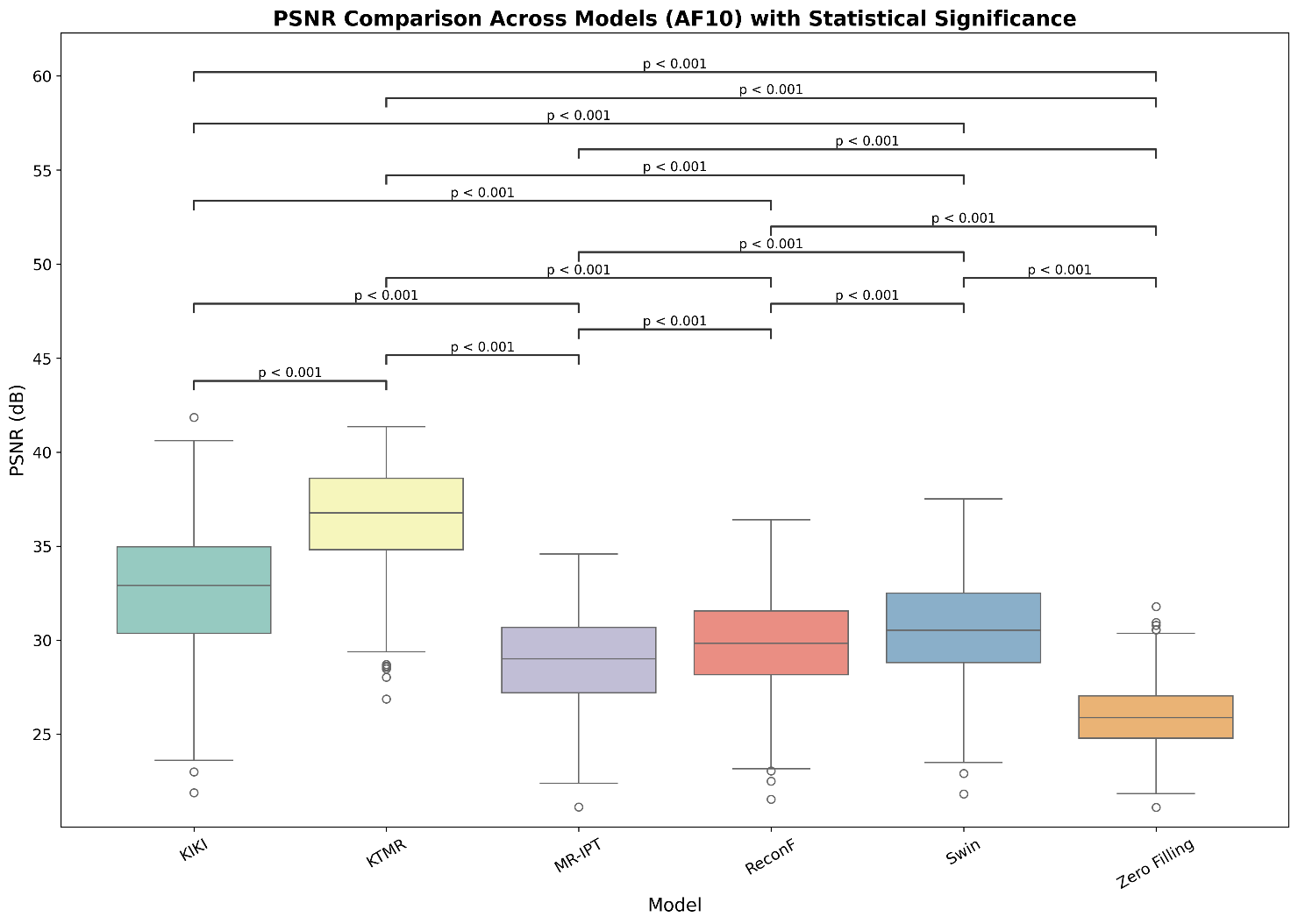


**Figure S8. PSNR comparison across all deep learning methods and zero-filled reconstruction at acceleration factor 10 (AF10).** Boxplots display the distribution of Peak Signal-to-Noise Ratio (PSNR) values in decibels (dB), where higher values indicate superior reconstruction fidelity. At this most aggressive acceleration level (only 10% of original data), KTMR (hybrid CNN-transformer) demonstrates exceptional robustness with the highest median PSNR (~36 dB), maintaining clinically viable reconstruction quality even under extreme undersampling. KIKI-net (pure CNN) achieves solid second-place performance (~32 dB), significantly outperforming all pure transformer methods (p < 0.001). Pure transformer approaches show substantial degradation: SwinMR (~30 dB), ReconFormer (~30 dB), and MR-IPT (~29 dB) cluster together with relatively similar performance levels, all significantly worse than the hybrid and CNN methods. Zero-filled reconstruction exhibits severely compromised quality (~26 dB). Statistical significance brackets indicate p-values from post-hoc Dunn's test with Benjamini-Hochberg correction following the Friedman test. The results demonstrate that KTMR's hybrid architecture maintains remarkable stability across all acceleration factors, with only 4.5% PSNR degradation from AF3 to AF10, while pure transformer methods suffer more dramatic quality losses, emphasizing the critical importance of CNN components for extreme acceleration scenarios in HP ¹²⁹Xe MRI reconstruction.


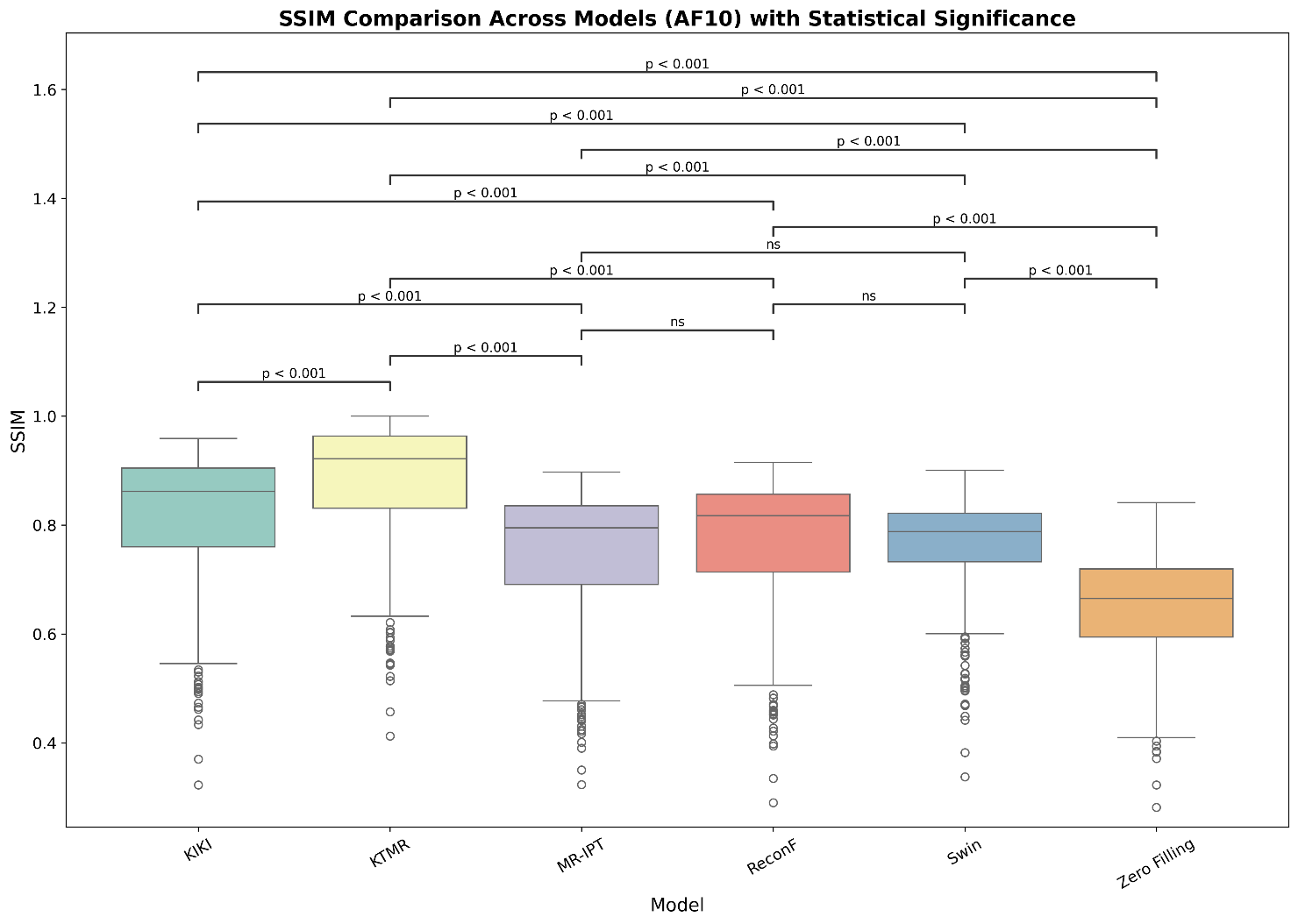


**Figure S9. SSIM comparison across all deep learning methods and zero-filled reconstruction at acceleration factor 10 (AF10).** Boxplots display the distribution of Structural Similarity Index Measure (SSIM) values, where higher values (closer to 1.0) indicate superior perceptual reconstruction quality and structural preservation. At this most extreme acceleration level (only 10% of original data), KTMR (hybrid CNN-transformer) maintains exceptional structural fidelity with the highest median SSIM (~0.88), demonstrating remarkable robustness under severe undersampling conditions. KIKI-net (pure CNN) achieves solid second-place performance (~0.82), with both top performers significantly outperforming all other methods (p < 0.001). Among pure transformer methods, MR-IPT (~0.75) and ReconFormer (~0.77) show non-significant differences (ns) between each other, while SwinMR (~0.76) performs similarly, with some non-significant differences (ns) noted between transformer approaches. Zero-filled reconstruction exhibits severely compromised structural similarity (~0.65). Statistical significance brackets indicate p-values from post-hoc Dunn's test with Benjamini-Hochberg correction following the Friedman test. These results demonstrate that KTMR's hybrid architecture maintains superior structural preservation capabilities even under the most challenging reconstruction scenarios, while pure transformer methods show substantial structural quality degradation, confirming the critical importance of CNN components for maintaining perceptual fidelity in extreme acceleration HP ¹²⁹Xe MRI reconstruction.

| Comparison | Acceleration Factor | Metric | P-Value |
| --- | --- | --- | --- |
| KTMR vs. ReconFormer | 3 | All metrics | < 0.001 |
| KTMR vs. SwinMR | 3 | All metrics | < 0.001 |
| KTMR vs. MR-IPT | 3 | All metrics | < 0.001 |
| KTMR vs. KIKI-net | 3 | PSNR | 0.001 |
| KTMR vs. KIKI-net | 3 | SSIM | < 0.001 |
| KTMR vs. KIKI-net | 3 | NMSE | 0.536 |
| KIKI-net vs. ReconFormer | 3 | All metrics | < 0.001 |
| KIKI-net vs. SwinMR | 3 | All metrics | < 0.001 |
| KIKI-net vs. MR-IPT | 3 | All metrics | < 0.001 |
| All DL vs. Zero Filling | 3 | All metrics | < 0.001 |
|  |  |  |  |
| KTMR vs. ReconFormer | 7 | All metrics | < 0.001 |
| KTMR vs. SwinMR | 7 | All metrics | < 0.001 |
| KTMR vs. MR-IPT | 7 | All metrics | < 0.001 |
| KTMR vs. KIKI-net | 7 | PSNR, SSIM | < 0.001 |
| KTMR vs. KIKI-net | 7 | NMSE | 0.03 |
| KIKI-net vs. ReconFormer | 7 | All metrics | < 0.001 |
| KIKI-net vs. SwinMR | 7 | All metrics | < 0.001 |
| KIKI-net vs. MR-IPT | 7 | All metrics | < 0.001 |
| MR-IPT vs. SwinMR | 7 | PSNR | 0.288 |
| MR-IPT vs. SwinMR | 7 | NMSE | 0.836 |
| MR-IPT vs. ReconFormer | 7 | SSIM | 0.007 |
| All DL vs. Zero Filling | 7 | All metrics | < 0.001 |
|  |  |  |  |
| KTMR vs. ReconFormer | 10 | All metrics | < 0.001 |
| KTMR vs. SwinMR | 10 | All metrics | < 0.001 |
| KTMR vs. MR-IPT | 10 | All metrics | < 0.001 |
| KTMR vs. KIKI-net | 10 | All metrics | < 0.001 |
| KIKI-net vs. ReconFormer | 10 | All metrics | < 0.001 |
| KIKI-net vs. SwinMR | 10 | All metrics | < 0.001 |
| KIKI-net vs. MR-IPT | 10 | All metrics | < 0.001 |
| MR-IPT vs. ReconFormer | 10 | NMSE | 0.008 |
| MR-IPT vs. SwinMR | 10 | SSIM | 0.137 |
| ReconFormer vs. SwinMR | 10 | SSIM | 0.338 |
| All DL vs. Zero Filling | 10 | All metrics | < 0.001 |

Table S1. Pairwise Statistical Comparisons of Reconstruction Performance Across Deep Learning Architectures
